# Supplementary material for: Circulating miRNAs for glioblastoma monitoring: from biofluid to clinical decision
Source: Mol Ther Oncol. 2026 Jul 28;34(3):201308. doi: 10.1016/j.omton.2026.201308 (PMC13476693; doi:10.1016/j.omton.2026.201308)
Supplement: Supplementary file 1 [file mmc1.pdf]

OMTON, Volume *34*

Supplemental information

Circulating miRNAs for glioblastoma monitoring: from biofluid to clinical decision

Arthur Leclerc, Evelyne Emery, and Guénaëlle Levallet

Table S1. Ranked summary of the most consistently validated circulating miRNAs in glioma across the studies included in this review. Supporting studies refer to independent peer-reviewed reports cited in the present review supporting the indicated clinical use of the miRNA in glioma; a higher count reflects greater inter-study reproducibility. ↑ = upregulated in glioma; ↓ = downregulated; Dynamic = variable longitudinal pattern tracking disease activity. CSF, cerebrospinal fluid; MGMT, O^6-methylguanine-DNA methyltransferase. Reference numbers correspond to the main manuscript reference list.

miRNA	Regulation	Biofluid(s)	Main clinical application(s)	Supporting studies	Consistency tier
**miR-21**	↑	Serum, plasma, CSF, exosomes	Diagnostic, prognostic, monitoring	13 (refs 41, 76–82, 112, 113, 123, 124, 126)	High
**miR-222**	↑	Serum, plasma, CSF	Prognostic, monitoring	4 (refs 98, 112, 114, 123)	Moderate–high
**miR-181 family**	↓	Serum, plasma	Prognostic, MGMT correlation, treatment response	3 (refs 83, 103, 117)	Moderate–high
**miR-182**	↑	Plasma, serum	Diagnostic, prognostic	3 (refs 86, 87, 114)	Moderate–high
**miR-124**	↓	Plasma, CSF	Diagnostic	3 (refs 89, 98, 112)	Moderate–high
**miR-128**	↓	Plasma, CSF	Diagnostic	3 (refs 79, 89, 98)	Moderate–high
**miR-210**	↑	Serum, plasma	Diagnostic, prognostic, anti-angiogenic monitoring	2 (refs 84, 85)	Limited
**miR-10b**	↑	CSF, serum	Diagnostic, monitoring	2 (refs 41, 78)	Limited
**miR-34a/b**	↓	Serum, plasma	Diagnostic	2 (refs 101, 102)	Limited
**miR-196b**	↑	CSF	Diagnostic, prognostic	1 (ref 41)	Single-study
**miR-137**	↓	Plasma	Prognostic	1 (ref 99)	Single-study
**miR-100**	↓	Plasma	Prognostic	1 (ref 100)	Single-study
**miR-223**	Dynamic	Serum	Longitudinal monitoring; recurrence vs. pseudo-progression	1 (ref 126)	Single-study
**miR-320e**	Dynamic	Serum	Longitudinal monitoring; recurrence vs. pseudo-progression	1 (ref 126)	Single-study
**miR-1238 (exosomal)**	↑	Exosomes	Temozolomide resistance	1 (ref 125)	Single-study
