# Supplementary material for: Circulating miRNAs for glioblastoma monitoring: from biofluid to clinical decision
Source: Mol Ther Oncol. 2026 Jul 28;34(3):201308. doi: 10.1016/j.omton.2026.201308 (PMC13476693; doi:10.1016/j.omton.2026.201308)
Supplement: Supplementary file 2 [file mmc2.pdf]

Molecular Therapy
Oncology
Review

Circulating miRNAs for glioblastoma monitoring: from biofluid to clinical decision

Arthur Leclerc,[1,2] Evelyne Emery,[2,3] and Guénaëlle Levallet[1,4]

[1]Université de Caen Normandie, CNRS, Normandie Université, ISTCT UMR6030, GIP CYCERON, 14000 Caen, France; [2]Centre Hospitalier Universitaire de Caen, Service de Neurochirurgie, 14000 Caen, France; [3]INSERM, U1237, PhIND "Physiopathology and Imaging of Neurological Disorders," Institut Blood and Brain @ Caen-Normandie, Cyceron, 14000 Caen, France; [4]Centre Hospitalier Universitaire de Caen, Service d'Anatomie et Cytologie Pathologiques, 14000 Caen, France

Gliomas, particularly glioblastoma (GBM), remain highly lethal brain tumors with limited treatments. MRI-based monitoring lacks specificity in distinguishing progression from treatment effects. Circulating microRNAs (miRNAs), small non-coding RNAs involved in tumor biology, show promise as liquid biopsy biomarkers. We searched PubMed, Embase, Scopus, and Web of Science for clinical studies investigating circulating miRNAs as diagnostic, prognostic, or predictive biomarkers in glioma, across plasma, serum, and cerebrospinal fluid (CSF). Oncogenic miRNAs (oncomiRs) such as miR-21, miR-10b, miR-182, and miR-210 were consistently upregulated in glioma patients, whereas tumor suppressors including miR-124, miR-128, miR-137, and miR-181 family members were downregulated. Prognostically, elevated miR-21, miR-222, and miR-196b correlated with poor survival, while reduced miR-181d, miR-100, and miR-145 predicted worse outcomes. Composite signatures, particularly exosomal or CSF-derived panels, outperformed single markers. Dynamic changes in miR-21, miR-222, and miR-210 reflected treatment response or progression, with exosomal miR-1238 emerging as a marker of temozolomide resistance. Longitudinal serum levels of miR-223 and miR-320e correlated with MRI tumor volume and distinguished true progression from pseudo-progression. Circulating miRNAs show strong potential as minimally invasive biomarkers for glioma management, but clinical translation remains limited by technical and biological variability and lack of validation. Advances in biosensors, multi-omics, and standardization are key to clinical integration.

INTRODUCTION

Gliomas, the most prevalent primary brain tumors, pose significant clinical challenges due to their infiltrative nature, histological heterogeneity, and propensity for recurrence.[1] Their classification, based on histopathological and molecular features, guides treatment decisions; however, the dynamic nature of these tumors often leads to therapeutic resistance and disease progression.[2] Current clinical management relies heavily on magnetic resonance imaging (MRI) for tumor surveillance, yet MRI has well-recognized limitations in differentiating treatment-related changes from true tumor progression, often leading to ambiguous interpretations and the need for invasive procedures.[3] Histological analysis, the cornerstone of glioma diagnosis, requires neurosurgery to obtain tumor tissue, presenting risks and discomfort to patients while failing to capture the full spatial and temporal heterogeneity of the tumor.[4] These limitations underscore the urgent need for non-invasive, reliable biomarkers that can accurately reflect tumor status, predict treatment response, and detect early signs of recurrence.[5]

The concept of liquid biopsy originated from the description by Mandel and Métais in 1948 of cell-free nucleic acids in human plasma,[6] but its translational potential emerged only decades later with the detection of tumor-derived DNA in the bloodstream of cancer patients.[7] Early clinical applications focused on hematological malignancies, where peripheral blood directly reflects tumor burden and allows sampling of malignant clones for diagnosis and minimal residual disease assessment.[8] Over the past two decades, the approach has expanded to solid organ malignancies, with circulating tumor DNA, circulating tumor cells, and extracellular vesicles becoming clinically actionable analytes in breast, lung, colorectal, and prostate cancers.[9] The application of liquid biopsy to central nervous system disorders is more recent and faces specific challenges, notably the restricted exchange of biomolecules across the blood-brain barrier (BBB). Nevertheless, the use of cerebrospinal fluid (CSF) as a tumor-proximal compartment, together with growing interest in liquid biopsy for non-neoplastic CNS conditions such as neurodegenerative diseases and traumatic brain injury,[10] has progressively positioned the field for clinical translation in neuro-oncology.

Liquid biopsy—the analysis of circulating tumor-derived material in easily accessible biofluids—holds immense promise in neuro-oncology by offering a minimally invasive approach to monitor disease dynamics and personalize treatment strategies.[11] It enables the detection of various tumor-derived components, including circulating tumor cells, cell-free DNA, circulating microRNAs (miRNAs), extracellular vesicles (EVs), proteins, and tumor-educated

https://doi.org/10.1016/j.omton.2026.201308.

Correspondence: Arthur Leclerc, Normandie University, UNICAEN, CNRS, ISTCT Unit, GIP CYCERON, 14000 Caen, France.
E-mail: arthur.leclerc@neurochirurgie.fr

Published by Elsevier Inc. on behalf of The American Society of Gene and Cell Therapy.
This is an open access article under the CC BY license (http://creativecommons.org/licenses/by/4.0/).

platelets.[12] Among these, circulating miRNAs have emerged as particularly promising biomarkers due to their stability in biofluids, cancer-specific expression patterns, and ease of detection.

Circulating miRNAs are small non-coding RNAs of approximately 18–22 nucleotides that regulate gene expression post-transcriptionally and exhibit remarkable stability in plasma, serum, CSF, and exosomes, owing to protection by RNA-binding proteins or encapsulation within vesicles.[13] The dysregulation of specific miRNAs has been implicated in glioma initiation, progression, and treatment resistance, supporting their potential as biomarkers reflecting tumor biology in real time.[14]

This review assesses current research on circulating miRNAs as tools for clinical monitoring in glioma patients, covering their potential use in diagnosis, prognosis, treatment response assessment, and differentiation of recurrence from radiation necrosis. It also provides a comprehensive overview of circulating miRNA biology, detection methods and a systematic analysis of clinically relevant miRNA candidates, highlighting the challenges and opportunities in translating these biomarkers into clinical practice.

RESULTS

Biological and technical background

miRNAs: From molecular function to cancer biology

miRNAs are evolutionarily conserved small non-coding RNAs of approximately 18–22 nucleotides that act as central post-transcriptional regulators of gene expression by guiding the RNA-induced silencing complex to partially complementary sequences in the 3′ untranslated regions of target messenger RNAs, leading to either translational repression or transcript degradation.[15,16] Each miRNA can regulate hundreds of distinct targets, and most protein-coding genes are themselves subject to combinatorial control by multiple miRNAs, generating dense regulatory networks that govern fundamental processes including proliferation, differentiation, apoptosis, metabolism, and stress responses.[17] Recent advances using structural biology, single-molecule imaging, and high-throughput functional screens have refined our understanding of the core biogenesis machinery, including the Microprocessor (Drosha-DGCR8) and Dicer complexes, and have revealed additional layers of regulation such as RNA editing, miRNA tailing, and target-directed miRNA decay.[18]

The relevance of miRNAs to oncology emerged from the seminal demonstration that the miR-15a/16-1 cluster is frequently deleted in chronic lymphocytic leukemia, establishing miRNAs as *bona fide* cancer genes.[19] Subsequent large-scale profiling work showed that virtually all human cancers display characteristic miRNA expression patterns, often allowing accurate tumor classification by miRNA signatures alone.[20] Functionally, miRNAs operate as oncogenes (oncogenic miRNAs [oncomiRs]) when their direct targets encode tumor suppressors and, conversely, as tumor suppressors when they silence oncogenic drivers.[21] The miR-17-92 cluster typifies the context-dependent nature of these effects, behaving either as an oncogene or as a tumor suppressor depending on cellular background and tissue of origin.[22] miR-21, one of the most extensively investigated oncomiRs, is upregulated across breast, lung, colorectal, prostate, and pancreatic cancers, where it represses pro-apoptotic targets such as PTEN and PDCD4 to promote proliferation, invasion, and resistance to therapy.[23] Together with the let-7 family, miR-34a, miR-155, and the miR-200 family, these molecules collectively influence virtually all hallmarks of cancer.

Beyond their intracellular regulatory function, miRNAs are released into the extracellular space within EVs, bound to RNA-binding proteins, or packaged in lipoprotein complexes, conferring remarkable stability in biofluids and positioning circulating miRNAs as attractive minimally invasive biomarkers across a broad spectrum of cancers.[24] The translation of this biology toward clinical applications has progressed along two parallel tracks: as diagnostic and prognostic biomarkers in liquid biopsy approaches and as therapeutic targets through miRNA mimics, antagomirs, and modified oligonucleotides, several of which have advanced to early-phase clinical trials in oncology.[25]

Biology of miRNAs in glioma pathophysiology

miRNA biogenesis is a multistep process beginning with transcription of primary miRNA transcripts (pri-miRNAs) by RNA polymerase II in the nucleus. These are processed by the Drosha-DGCR8 complex into precursor hairpins (pre-miRNAs), exported to the cytoplasm via Exportin-5, and cleaved by Dicer into ∼22-nucleotide duplexes. The guide strand is incorporated into the RNA-induced silencing complex (RISC), mainly bound to Argonaute proteins, enabling sequence-specific binding to the 3′ untranslated regions (UTRs) of target mRNAs and leading to translational repression or degradation.[15,16]

Gliomas exhibit extensive miRNA dysregulation, occurring through genetic alterations (e.g., copy number variations), epigenetic modifications (e.g., CpG island hypermethylation, histone deacetylation), and altered signaling pathways affecting miRNA processing.[14,26–29] This results in a profound imbalance between oncomiRs and tumor-suppressor miRNAs, both contributing to tumor initiation and progression.

Several miRNAs act as tumor suppressors. miR-7 and miR-128 are typically downregulated in glioblastoma (GBM), leading to unchecked activation of the EGFR/PI3K/AKT and E2F3a pathways, respectively, promoting proliferation and angiogenesis.[30,31] Similarly, miR-34a induces apoptosis and suppresses migration but is consistently reduced in GBM tissue.[32,33] Conversely, oncomiRs are upregulated: miR-21 promotes proliferation, invasion, and resistance to apoptosis by targeting PTEN, PDCD4, and TPM1[34–36]; miR-10b facilitates invasion and angiogenesis through regulation of HOXD10, RhoC, and uPAR[37]; and miR-93 and miR-221/222 contribute to angiogenesis, cell cycle progression, and radiotherapy resistance.[14,38]

Altogether, miRNAs exert pleiotropic functions in GBM pathophysiology, affecting virtually all hallmarks of cancer. Their dynamic

deregulation highlights both their potential as biomarkers for disease monitoring and as therapeutic targets.[39,40]

Circulation and stability in biofluids

Circulating miRNAs are detectable in plasma, serum, and CSF.[41,42] Their presence arises from active secretion via EVs—particularly exosomes and microvesicles—which protect them from enzymatic degradation[43] and from passive release during apoptosis and necrosis.[44] Stability is conferred by vesicular protection, association with RNA-binding proteins such as Argonaute-2, or encapsulation within lipoprotein complexes.[45,46] These properties make circulating miRNAs consistently detectable in clinical specimens, as summarized in Table 1.

When comparing biofluids, three patterns emerge: serum typically shows higher apparent miRNA levels than plasma due to platelet activation during coagulation[47,49,53]; CSF has lower RNA biomass yet hundreds of miRNAs remain detectable, with only moderate concordance between CSF and blood profiles,[50] and small-RNA composition differs across biofluids and between bulk versus EV-enriched fractions.[51,55–57] Longitudinal work shows that, under standardized platelet-free plasma workflows, many miRNAs are highly stable over months, though technical variability dominates if extraction is not tightly controlled.[48,53,54] Practically, platelet-free plasma is the most reproducible choice, serum is usable if coagulation is standardized, and CSF demands larger input volumes and low-input-optimized protocols.[50,52,53,58]

Pre-analytical variability

Pre-analytical variables—sample collection, processing, storage, and RNA extraction—significantly impact the accuracy and reproducibility of circulating miRNA measurements.[59] The choice of anticoagulant, venipuncture technique, and centrifugation speed directly affect plasma and serum composition.[60] Circulating miRNAs remain stable in whole blood for up 48 h.

Key recommendations for miRNA quality preservation include use of EDTA-containing collection tubes with processing within 4 h,[61] storage at -80°C,[61] minimization of freeze-thaw cycles,[61,62] use of extraction kits specifically designed for small RNAs,[61] and harmonization of all pre-analytical steps including the use of exogenous spike-ins.[61] Specialized stabilization tubes (e.g., Streck Nucleic Acid BCT) can maintain cell-free RNA/miRNA concentrations for up to 7 days at ambient temperature.[63] Contamination with white blood cells from inflammatory processes should be considered during interpretation.[64]

Detection methods

Several platforms are available for circulating miRNA quantification, each with distinct advantages (Table 2). Real-time RT-qPCR offers high sensitivity, specificity, and dynamic range for absolute quantification of small miRNA panels.[66,67] Microarray platforms enable high-throughput profiling of hundreds to thousands of miRNAs simultaneously using immobilized complementary probes.[68,69] Next-generation sequencing (NGS; small RNA sequencing [RNA-seq]) provides comprehensive, unbiased detection of both known and novel miRNAs.[70,71] The selection of detection platform should align with the study objective: RT-qPCR for focused validation, microarrays for large-scale discovery, and NGS for comprehensive profiling.

RT-qPCR

RT-qPCR remains the most widely used method for focused validation of circulating miRNA biomarkers, offering high sensitivity, specificity, and dynamic range for absolute quantification of small miRNA panels.[66,67] It has been the technique of choice for clinical translation studies in solid malignancies, as illustrated by a recent multicenter European low-dose CT screening program in which a nine-miRNA plasma signature was deployed by RT-qPCR for early detection of lung cancer with consistent diagnostic accuracy across cohorts.[72] Its main limitations include restricted multiplexing capacity and a dependence on prior sequence knowledge.

Microarrays

Microarray platforms enable high-throughput profiling of hundreds to thousands of miRNAs simultaneously using immobilized complementary probes, providing semi-quantitative expression maps useful for hypothesis-generating studies.[68,69] They have been extensively applied to identify miRNA signatures in breast, lung, and colorectal cancers but are gradually being superseded by sequencing-based approaches given concerns regarding cross-hybridization and reduced sensitivity for low-abundance miRNAs.[73]

NGS (small RNA-seq)

NGS of small RNAs provides comprehensive, unbiased detection of both known and novel miRNAs, including isomiRs, sequence variants, and other small non-coding RNA species.[70,71] Small RNA-seq has been instrumental in characterizing the complete circulating miRNA landscape across biofluids and has been widely applied across solid malignancies for biomarker discovery; however, the higher cost, the requirement for specialized bioinformatic pipelines, and the need for careful normalization currently limit its routine clinical use.

Droplet digital PCR

Droplet digital PCR (ddPCR) represents an emerging quantitative platform offering absolute measurement of low-abundance circulating miRNAs without reliance on a calibration curve, with high precision and improved tolerance to inhibitors commonly present in plasma and serum.[74] ddPCR has been successfully applied to circulating miRNA quantification in ovarian, breast, and lung cancers and is increasingly attractive for monitoring minimal residual disease and dynamic biomarker variations during treatment.

Electrochemical biosensors and emerging platforms

A range of biosensor formats—including electrochemical, plasmonic, and microfluidic devices—is under active development to address the unmet need for rapid, low-cost, and point-of-care

Table 1. Comparison of biofluids for circulating miRNA analysis

Axis	Plasma	Serum	CSF	Key references
Relative miRNA load/abundance	lower than serum in paired comparisons; sensitive to platelet depletion (prefer PFP)	typically higher apparent levels than plasma, partly from platelet release during coagulation	much lower RNA biomass overall; detectable but low abundance	Wang, 2012[47]; Sandau, 2024[48]; Dufourd, 2019[49]; Burgos, 2013[50]
Detected miRNA repertoire (NGS)	not directly reported in these specific comparisons	∼532 miRNAs detected (example dataset)	∼486 miRNAs detected; 353 shared with serum; distinct profile	Burgos, 2013[50]
Cross-matrix correlations	–	high inter-subject correlation (∼0.85)	high inter-subject correlation (∼0.87); within-subject CSF–blood median Spearman ≈0.60	Burgos, 2013[50]
Small-RNA class composition	high miRNA-to-tDR ratio among biofluids (e.g., ∼72)	similar classes to plasma but shifted by coagulation effects	composition differs from blood; lower mapping rates common	Godoy, 2018[51]; Burgos, 2013[50]; Alsop, 2022[52]
Longitudinal stability (healthy)	many miRNAs stable over months under standardized PFP workflows; technical steps can dominate variance if not controlled	expected stable if clotting is standardized, but more variable than plasma due to coagulation	not directly quantified; detectability highly protocol-dependent	Sandau, 2024[48]; Max, 2018[53]; Wang, 2012[47]; Burgos, 2013[50]
Typical input volumes for profiling	200–500 μL commonly sufficient (qPCR/NGS) with PFP	200–500 μL commonly used; document clotting conditions	≥1 mL typically needed for robust detection; low-input methods essential	Sandau, 2024[48]; Dufourd, 2019[49]; Burgos, 2013[50]
Pre-analytics to prioritize	double/triple centrifugation to obtain PFP; consistent venipuncture and handling	standardize clotting time/conditions; minimize platelet activation	avoid blood contamination; swift processing; concentrate RNA	Sandau, 2024[48]; Wang, 2012[47]; Burgos, 2013[50]
Major confounders	residual platelets; variable extraction/inhibition if workflows differ	coagulation/platelet release; platform biases across studies	extremely low input leading to adapter dimer and contaminant amplification; any blood carryover	Freedman, 2016[54]; Wang, 2012[47]; Alsop, 2022[52]; Burgos, 2013[50]

Key characteristics of plasma, serum, and CSF are compared across miRNA abundance, detection repertoire, longitudinal stability, and pre-analytical requirements. CSF, cerebrospinal fluid; miRNA, microRNA; NGS, next-generation sequencing; PFP, platelet-free plasma; qPCR, quantitative polymerase chain reaction; RNA, ribonucleic acid; tDR, transfer RNA-derived fragment.

Table 2. Analytical platforms for circulating miRNA detection: principles, advantages, and limitations

Method	Principle	Advantages	Limitations	Best use case/key references
RT-qPCR	reverse transcription of mature miRNAs into cDNA, followed by PCR amplification with specific primers (SYBR Green or TaqMan probes)	high sensitivity and specificity; quantitative (absolute or relative); relatively low cost and fast turnaround	limited to known miRNAs; susceptible to normalization bias; small panels only	validation of candidate miRNAs in clinical studies (Chen, 2021[36]; Kroh, 2010[65])
Microarray	hybridization of fluorescently labeled RNA to immobilized probes complementary to known miRNAs	high-throughput (hundreds to thousands of miRNAs); relatively low cost per sample; well established protocols	lower sensitivity than RT-qPCR or NGS; Limited to annotated miRNAs; Semi-quantitative	discovery of differential expression profiles in large cohorts (Godoy, 2018[51]; Wang, 2017)
NGS (small RNA-seq)	deep sequencing of barcoded small-RNA libraries generated from biofluids	comprehensive (detects both known and novel miRNAs); high resolution (isomiRs, isoforms); quantitative and unbiased	expensive and time-consuming; requires advanced bioinformatics; sensitive to library prep bias	discovery of new miRNAs, isoform analysis, complete profiling (O'Brien, 2018[16])

cDNA, complementary DNA; miRNA, microRNA; NGS, next-generation sequencing; PCR, polymerase chain reaction; RT-qPCR, quantitative reverse transcription PCR; RNA, ribonucleic acid; RNA-seq, RNA sequencing; SYBR, SYBR Green fluorescent dye.

miRNA detection. Recent electrochemical biosensors enhanced with nanomaterials have demonstrated highly sensitive and specific detection of disease-associated miRNAs in liver and pancreatic cancers, with attomolar detection limits reported in some configurations.[75,76] Although their clinical implementation remains limited, these platforms are particularly promising for serial monitoring scenarios such as post-operative surveillance in neuro-oncology.

Clinical applications of circulating miRNAs in glioma

Diagnostic biomarkers

Identifying reliable biomarkers for early GBM diagnosis remains challenging given the limited specificity of conventional neuroimaging. Numerous studies have evaluated the diagnostic potential of circulating miRNAs in plasma, serum, or CSF. The main candidates are summarized in Table 3.

miR-21 is the most extensively studied, consistently overexpressed in serum, plasma, and CSF exosomes of GBM patients. It acts as an oncomiR by repressing PTEN and PDCD4, promoting proliferation, invasion, and apoptosis resistance.[41,79–84,95,113] Isoform-specific analyses, such as enrichment of miR-21-3p in CD44+ EVs, suggest that vesicle subtypes may carry more discriminative signatures than bulk specimens.[113] Other upregulated oncomiRs include miR-10a/b, associated with stemness and invasion in CSF[41,83]; miR-210, a hypoxia-regulated miRNA reflecting tumor hypoxia and angiogenesis[100,101]; and miR-182, elevated in plasma and promoting proliferation via FOXO3 and BCL2.[98,99] Additional upregulated candidates include miR-23a-3p, miR-26a, miR-30e, miR-140, miR-195-5p, miR-29a-3p, miR-433-3p, miR-363, miR-582-5p, miR-574-3p, and exosomal miR-320.[41,77,80,85–87,89,103–105,109,110]

Conversely, several tumor-suppressor miRNAs are consistently downregulated: miR-124 and miR-128 (neuronal differentiation-associated, decreased in both plasma and CSF),[77,93] miR-137,[94] miR-100,[92] miR-34a/b (regulating EGFR and cell cycle checkpoints),[90,91] as well as miR-383, miR-29b, miR-485-3p/5p, and miR-181 family members.[77,81,88,96,108,114,115] Notably, CSF-derived miR-711 (upregulated) and miR-935 (downregulated) may differentiate GBM from medulloblastoma.[111] Some biomarkers show context-dependent expression: miR-1 has been reported as both increased and decreased,[77,78] miR-106a-5p appears enriched in CD44+ EVs but may function as a tumor suppressor,[113] and miR-451a acts as a metabolic rheostat via the LKB1/AMPK/CAB39 pathway.[106,107]

A recurring limitation is that single biomarkers often lack reproducibility across cohorts. Composite signatures have consistently demonstrated superior diagnostic accuracy: CSF-based panels combining let-7a/b, miR-10a/b, miR-30e, miR-140, and miR-196a/b yielded excellent sensitivity and specificity[41]; EV-based signatures including miR-21, miR-21-3p, miR-15b-3p, and miR-106a-5p improved discrimination[113]; and plasma- and serum-based panels further confirmed the added value of multimarker approaches.[89,105] These data suggest that miRNA signatures, rather than individual

Table 3. Circulating miRNAs with diagnostic potential in glioma patients

miR	Fluid	Expression vs. healthy	Cellular function (succinct)	Authors (name, year)
miR-1	plasma/serum	variable	invasion (TKS4/TKS5/EFHD2)	Herman, 2015[77]; Díaz Méndez, 2023[78]
let-7a	CSF	increased	tumor suppressor (context dependent in CSF panel)	Kopková, 2019[41]
let-7b	CSF	increased	tumor suppressor (context-dependent in CSF panel)	Kopková, 2019[41]
miR-10a	CSF	increased	stemness/invasion	Kopková, 2019[41]; Teplyuk, 2012[79]
miR-10b	CSF	increased	stemness/invasion	Teplyuk, 2012[79]; Kopková, 2019[41]
miR-15b-3p	serum (CD44+ EVs)	increased	no established function in glioma (diagnostic biomarker only)	Tzaridis, 2020[80]
miR-21	CSF (exosomes/EVs); serum/plasma	increased	OncomiR; targets PTEN/PDCD4; invasion	Akers, 2013[81]; Baraniskin, 2012[82]; Teplyuk, 2012[79]; Kopková, 2019[41]; Wang, 2012[83]; Parvizhamidi, 2018[84]; Tzaridis, 2020[80]
miR-21-3p	serum (CD44+ EVs)	increased	OncomiR	Tzaridis, 2020[80]
miR-23a-3p	plasma	increased	oncogene; invasion via HOXD10, PI3K/AKT	Herman, 2015[77]; Hu, 2013[85]; Yachi, 2018[86]
miR-26a	serum	increased	oncogene; targets PTEN	Parvizhamidi, 2018[84]; Huse, 2009[87]
miR-29b	serum (exosomes)	decreased	tumor suppressor; ECM regulation	Zhong, 2019[88]
miR-29a-3p	plasma	increased	proliferation suppressor	Géczi, 2021[89]
miR-30e	CSF	increased	cell cycle/apoptosis	Kopková, 2019[41]
miR-34a	serum	decreased	tumor suppressor; EGFR axis	Rahmati, 2021[90]
miR-34b-3p	plasma (exosomes)	increased	tumor suppressor	Yamashita, 2021[91]
miR-100	serum	decreased	suppresses proliferation/migration	Zhang, 2019[92]
miR-106a-5p	serum (CD44+ EVs)	variable	suppressor; targets SLC2A3, E2F1, FASTK	Tzaridis, 2020[80];
miR-124	plasma/CSF	decreased	tumor suppressor; neuronal differentiation	Herman, 2015[77]; Akers, 2017[93]
miR-128	plasma/CSF	decreased	tumor suppressor; neuronal identity	Herman, 2015[77]; Akers, 2017[93]
miR-137	serum	decreased	tumor suppressor; differentiation	Li, 2016[94]
miR-140	CSF	increased	migration/ECM regulation	Kopková, 2019[41]
miR-181d	plasma/serum (exosomes)	decreased	predictive of MGMT; treatment response	Sippl, 2022[95]; Stakaitis, 2020[96]
miR-181a	plasma	decreased	tumor suppressor	Wu, 2022[97]
miR-181b	plasma	decreased	tumor suppressor	Wu, 2022[97]
miR-182	plasma	increased	oncogene; targets FOXO3, BCL2	Xiao, 2016[98]; Kouri, 2015[99]
miR-195-5p	plasma	increased	cell cycle regulation	Géczi, 2021[89]
miR-196a	CSF	increased	oncogene; HOX gene regulation	Kopková, 2019[41]
miR-196b	CSF	increased	oncogene; poor prognosis	Kopková, 2019[41]
miR-210	serum; serum exosomes	increased	hypoxia oncomiR	Lai, 2015[100]; Lan, 2020[101]
miR-222	CSF; serum (exosomes)	increased	oncogene; invasion; DNA repair	Akers, 2017[93]; Olioso, 2021[102]
miR-302c-3p	plasma	increased		Herman, 2015[77]

(*Continued on next page*)

Table 3. *Continued*

miR	Fluid	Expression vs. healthy	Cellular function (succinct)	Authors (name, year)
			suppressor; inhibits proliferation/invasion (targets MTDH)	
miR-320	serum (exosomes)	increased	suppressor; inhibits SND1/β-catenin	Manterola, 2014[103]; Li, 2017[104]
miR-342-3p	plasma	Dysregulated	neuronal identity	Wang, 2012[83]
miR-363	serum	increased	diagnostic biomarker	Vojdani, 2021[105]
miR-383	plasma	decreased	suppressor; proliferation/invasion	Herman, 2015[77]
miR-433-3p	plasma	increased	neuronal development	Géczi, 2021[89]
miR-451a	serum	changed	metabolic rheostat; LKB1/AMPK via CAB39	Godlewski, 2010[106]; Kim, 2011[107]
miR-485-3p	serum	decreased	suppressor; inhibits RNF135	Wang, 2017[108]
miR-485-5p	serum	decreased	tumor-suppressor biomarker	Rahmati, 2021[90]
miR-574-3p	serum (microvesicles)	increased	biomarker; mechanistic role unclear	Manterola, 2014[103]; Jiang, 2018[109]; Peng, 2022[110]
miR-582-5p	serum	increased	diagnostic biomarker	Vojdani, 2021[105]
miR-711	CSF	increased	differential vs. medulloblastoma	Yang, 2013[111]
miR-935	CSF	decreased	differential vs. medulloblastoma	Yang, 2013[111]
miR-2276-5p	plasma (exosomes)	decreased	targets RAB13	Sun, 2021[112]
miR-4298	serum	changed	no function reported in glioma	Wang, 2017[108]

Expression changes are reported relative to healthy controls. AKT, protein kinase B; AMPK, AMP-activated protein kinase; BCL2, B cell lymphoma 2; CAB39, calcium-binding protein 39; CD44, cluster of differentiation 44; CSF, cerebrospinal fluid; ECM, extracellular matrix; EFHD2, EF-hand domain family member D2; EGFR, epidermal growth factor receptor; EV, extracellular vesicle; FASTK, Fas-activated serine/threonine kinase; FOXO3, forkhead box O3; HOX, homeobox; HOXD10, homeobox D10; LKB1, liver kinase B1; MGMT, O6-methylguanine-DNA methyltransferase; miR, microRNA; MTDH, metadherin; PDCD4, programmed cell death 4; PI3K, phosphoinositide 3-kinase; PTEN, phosphatase and tensin homolog; RAB13, Ras-related protein Rab-13; RNF135, ring finger protein 135; SLC2A3, solute carrier family 2 member 3; SND1, staphylococcal nuclease and Tudor domain containing 1; TKS4/TKS5, tyrosine kinase substrate 4/5.

miRNAs, represent the most reliable strategy for non-invasive GBM diagnosis.

Prognostic biomarkers

Circulating miRNAs have been extensively investigated as prognostic biomarkers, with several candidates showing correlations with overall survival (OS) and progression-free survival (PFS) (Table 4).

Among upregulated oncomiRs, miR-21 correlates with shorter OS and higher recurrence risk across serum, plasma, and CSF exosomes.[95,102,113,117] Other adverse prognostic markers include miR-222 (predicting reduced OS and disease-free survival [DFS]),[102,116] miR-210 (reflecting tumor hypoxia),[101] miR-182,[116] miR-196b,[41] miR-193b,[122] miR-20a-5p, and miR-106a-5p.[116] Exosomal signatures have revealed that high expression of miR-15b-3p, miR-21-3p, and miR-328-3p in CD44+ vesicles strongly associates with poor outcome,[113] and miR-221 and miR-155 are adverse prognostic biomarkers across multiple biofluids.[40,120]

Conversely, reduced levels of tumor-suppressor miRNAs correlate with poor prognosis. Decreased miR-181a, miR-181b, and miR-181d have been associated with shorter OS, with miR-181d also predicting MGMT methylation status and treatment response.[81,96,97] Other examples include miR-137,[94] miR-100,[92] miR-205,[124] miR-185,[121] miR-2276-5p,[112] miR-485-3p,[108] miR-29b,[88] miR-203, miR-663,[123] and miR-151a.[119] Interestingly, low circulating miR-30e has paradoxically been associated with better OS.[40] Composite signatures again outperform single miRNAs. A three-miRNA serum panel (miR-1-3p, miR-26a-1-3p, miR-487b-3p) stratified isocitrate dehydrogenase (IDH)-wild-type GBM patients and predicted survival,[78] while vesicle-derived panels (miR-15b-3p, miR-21-3p, miR-328-3p) demonstrated superior prognostic discrimination.[113] Integrated miRNA signatures hold the greatest promise for robust survival prediction.

Monitoring treatment response

Circulating miRNAs have been explored as dynamic biomarkers to monitor treatment response and disease progression. Several studies show that longitudinal changes in miRNA expression mirror therapeutic efficacy or herald recurrence (Table 5).

miR-21 levels typically decrease in responders following surgery, chemoradiation, or anti-angiogenic therapy but remain elevated or increase at progression.[83,102,125,126] miR-222 demonstrates a similar pattern,[102,125,126] and hypoxia-associated miR-210 decreases with effective therapy and re-elevates upon resistance, making it particularly relevant for bevacizumab monitoring.[101,126] miR-10b in CSF and serum marks active disease,[41] exosomal miR-124-3p rises in progressive disease,[102] and miR-1238 mediates temozolomide resistance—being enriched in exosomes from resistant patients and decreasing when sensitivity is restored.[127]

Several tumor-suppressor miRNAs correlate with treatment benefit: higher miR-181d predicts better response to chemoradiation, consistent with its association with MGMT methylation[81,96]; normalization of miR-485-3p after therapy associates with favorable response[108]; and changes in miR-128 and miR-342-3p have been noted across treatment courses.[83] Panel-based approaches improve discrimination between responders and non-responders.

These results underscore the potential of circulating miRNAs as real-time indicators of treatment response. However, reproducibility across cohorts remains limited, and composite panels measured longitudinally are likely to offer the most reliable monitoring tools.

Differentiating tumor recurrence from radionecrosis

Post-operative monitoring remains challenging, as MRI can be confounded by pseudo-progression or pseudo-response, particularly after chemoradiation or anti-angiogenic therapy.

The pivotal study by Morokoff et al. is, to date, the only systematic prospective investigation of serum miRNAs for post-operative glioma follow-up. In a cohort of 91 glioma patients (44 GBM, 47 low-grade glioma [LGG]) and 17 healthy controls, they identified a nine-miRNA serum signature with near-perfect accuracy for distinguishing glioma from healthy subjects. In longitudinal analysis of 11 patients with serial samples, miR-223 (in LGG) and miR-320e (in GBM) demonstrated dynamic fluctuations closely correlated with MRI-measured tumor volume. Critically, serum miRNA levels did not increase in cases of pseudo-progression—unlike MRI changes—suggesting that circulating miRNAs may provide additional specificity to distinguish true progression from treatment-related effects.[128] Combining miR-223, miR-320e, miR-21, and miR-23a achieved diagnostic accuracy approaching 100%, with miR-223 and miR-320e emerging as the most promising markers for longitudinal follow-up (Table 6).

Although limited by its relatively small longitudinal subset, this study provides proof of concept that serial monitoring of selected circulating miRNAs can complement MRI for post-operative surveillance. These findings strongly argue for larger multicentric validation studies.

Integration with established molecular markers

Contemporary glioma diagnosis and management rest on a molecular framework that has been substantially refined by the 2021 WHO Classification of Tumors of the Central Nervous System, in which IDH mutation status, 1p/19q codeletion, MGMT promoter methylation, and additional molecular features now define disease subtypes and inform therapeutic decisions.[129] Any liquid biopsy candidate biomarker must therefore be evaluated against this tissue-based reference framework to determine whether it provides complementary or redundant information.

IDH mutation status. Although IDH mutations are detectable in plasma cell-free DNA in a subset of patients, this assay shows limited sensitivity in GBM. Several circulating miRNA signatures have been reported to differ according to IDH status; in particular, a three-miRNA

Table 4. Circulating miRNAs with prognostic value in glioma

miR	Fluid/matrix	Variation associated with prognosis	Prognosis	Notes	Authors (name, year)
miR-1-3p	serum	low → poor OS/PFS	poor	part of 3-miR serum signature (with miR-26a-1-3p, miR-487b-3p); lower levels stratify IDHwt and associate with worse survival	Díaz Méndez, 2023[78]
miR-10b	CSF/serum	high → poor OS	poor	high CSF levels associate with median OS ∼9 vs. 16.5 months (low)	Kopková, 2019[41]
miR-15b-3p	serum EV (CD44+)	high → poor outcome	poor	increased in EVs; linked to poor survival	Tzaridis, 2020[80]
miR-20a-5p	serum	high → poor DFS	poor	high levels associate with decreased survival	Zhao, 2017[116]
miR-21	serum/CSF exosomes; EVs	high → poor OS/recurrence	poor	OncomiR; elevated levels correlate with shorter OS and higher recurrence risk	Olioso, 2021[102]; Shi, 2015[117]; Tzaridis, 2020[80]; Joo Park, 2022[113]
miR-21-3p	serum EV (CD44+)	high → poor outcome	poor	isoform enriched in EVs; associated with adverse outcome	Tzaridis, 2020[80]
miR-26a-1-3p	serum	low → poor OS/PFS	poor	with miR-1-3p & miR-487b-3p in serum signature; lower = worse	Díaz Méndez, 2023[78]
miR-29b	serum (exosomes)	low → poor OS	poor	underexpressed in GBM; low exosomal levels predict shorter survival; increase post-surgery	Zhong, 2019[88]
miR-100	serum	low → poor outcome	poor	lower serum miR-100 predicts worse outcome	Zhang, 2019b[92]
miR-106a-5p	serum	high → poor OS/DFS	poor	high levels associate with poorer outcomes	Zhao, 2017[116]
miR-137	serum	low → poor prognosis	poor	decreased in GBM; lower levels predict poorer prognosis	Li, 2016[94]
miR-145-5p	serum	high → better OS/DFS	Better	higher serum miR-145-5p independently predicts improved survival	Zhang, 2019a[118]; Zhao, 2017[116]
miR-145-5p	serum	low → poor OS/DFS	poor	independent prognostic indicator; lower level associates with worse survival	Zhang, 2019a[118]; Zhao, 2017[116]
miR-151a	CSF exosomes/serum	low → poor OS/PFS (TMZ-treated)	poor	lower expression associated with worse survival; restoration sensitizes to TMZ	Zeng, 2018[119]
miR-155	serum/plasma/CSF	high → poor outcome	poor	elevated levels linked to adverse prognosis	Bustos, 2022[120]; Ordoñez-Rubiano, 2024[40]
miR-181b	plasma	low → poor prognosis	poor	lower concentrations associate with worse outcome; inverse relation with functional scores	Wu, 2022[97]; Stakaitis, 2020[96]
miR-181d	plasma/serum; tumor	low → poor prognosis	poor	lower blood levels associate with worse prognosis; also predictive of MGMT/treatment response	Sippl, 2022[95]; Stakaitis, 2020[96]
miR-181a	plasma	low → poor prognosis	poor	lower concentrations associate with worse outcome	Wu, 2022[97]
miR-182	serum	high → poor OS/DFS	poor	high pre-treatment serum levels associate with poor prognosis	Zhao, 2017[116]
miR-185	plasma	low → poor OS	poor	decline in plasma level associated with poor survival; glioma-specific vs. other CNS tumors	Tang, 2015[121]
miR-193b	serum	high → poor OS	poor	promotes proliferation/invasion; high predicts poor survival	Zhu, 2019[122]
miR-196b	CSF/serum	high → poor OS	poor	overexpressed; poor survival association	Kopková, 2019[41]
miR-203	serum	low → poor OS/PFS	poor	reduced circulating miR-203 predicts worse OS/PFS	Chen, 2017b[123]

(*Continued on next page*)

Table 4. Continued

miR	Fluid/matrix	Variation associated with prognosis	Prognosis	Notes	Authors (name, year)
miR-205	serum	low → poor OS/PFS	poor	decreased levels associated with worse survival	Yue, 2016[124]
miR-210	serum/Exosomes	high → poor OS	poor	hypoxia-related; high levels predict poor survival	Lan, 2020[101]
miR-221	serum/plasma/CSF	high → poor outcome	poor	elevated in liquid biopsies; associated with prognosis	Bustos, 2022[120]; Ordoñez-Rubiano, 2024[40]
miR-222	serum (total/exosomal); CSF	high → poor OS/DFS	poor	OncomiR; higher levels predict decreased survival	Olioso, 2021[102]; Zhao, 2017[116]
miR-328-3p	serum EV (CD44+)	high → poor outcome	poor	included among increased EV-miRNAs linked to poor survival	Tzaridis, 2020[80]
miR-485-3p	serum	low → poor OS	poor	low serum level predicts shorter survival	Wang, 2017[108]
miR-487b-3p	serum	low → poor OS/PFS	poor	with miR-1-3p & miR-26a-1-3p in serum signature; lower = worse	Díaz Méndez, 2023[78]
miR-663	serum/tissue	low → poor outcome	poor	lower miR-663 associated with unfavorable clinical outcome	Chen, 2017b[123]
miR-2276-5p	plasma (exosomes)	low → poor OS	poor	targets RAB13; low in exosomes associates with higher grade and shorter survival	Sun, 2021[112]

Associations with OS, PFS, and DFS are reported. CD44, cluster of differentiation 44; CNS, central nervous system; CSF, cerebrospinal fluid; DFS, disease-free survival; EV, extracellular vesicle; GBM, glioblastoma; IDHwt, isocitrate dehydrogenase wild-type; MGMT, O6-methylguanine-DNA methyltransferase; miR, microRNA; OS, overall survival; PFS, progression-free survival; RT, radiotherapy; TMZ, temozolomide.

serum panel (miR-1-3p, miR-26a-1-3p, miR-487b-3p) was specifically deregulated in IDH-mutant compared with IDH-wild-type gliomas and retained independent prognostic value.[78] Such signatures may complement direct IDH genotyping by capturing downstream biological effects of IDH mutation, including the metabolic reprogramming triggered by 2-hydroxyglutarate accumulation.

MGMT promoter methylation. MGMT promoter methylation predicts temozolomide benefit but is assayed on tumor tissue, which is not always available, particularly at recurrence. miR-181d has emerged as the most consistently reported circulating biomarker correlated with MGMT methylation status and chemotherapy benefit,[81,96] and the broader landscape of miRNAs regulating MGMT expression and modulating temozolomide sensitivity has been systematically reviewed.[130] Circulating miR-181d levels could therefore serve as a surrogate marker when tumor tissue is unavailable.

Molecular subtypes. Beyond IDH and MGMT, GBM displays transcriptomic and methylation-based subtypes (proneural, classical, mesenchymal) with distinct biological behaviors and therapeutic vulnerabilities. Several miRNAs are differentially expressed across these subtypes—for instance, miR-21 is enriched in mesenchymal tumors, whereas miR-128 is downregulated in proneural tumors—suggesting that subtype-specific circulating signatures may eventually be defined to refine non-invasive glioma classification.[131]

Independent versus complementary value. Whether circulating miRNAs provide truly independent prognostic information beyond IDH status, MGMT methylation, and clinical variables remains an open question. Both the Morokoff serum signature[128] and the IDH-stratified three-miRNA panel[78] retained predictive value in multivariable analyses, suggesting that circulating miRNAs contribute complementary rather than purely redundant information. Nevertheless, head-to-head comparisons with established molecular markers in large prospective cohorts are still lacking and represent a priority for future translational efforts.

To enable a semi-quantitative comparison across studies, Table S1 ranks the circulating miRNAs most consistently validated in glioma in the present review, stratified by the number of independent supporting studies. miR-21 emerges as the most reproducible candidate, followed by miR-222, miR-210, the miR-181 family, miR-182, and miR-10b. By contrast, several promising candidates, including miR-223, miR-320e, and the exosomal miR-1238, currently rest on single high-quality studies and warrant independent validation before clinical adoption.

Synthesis

Figure 1 provides an integrative overview of circulating miRNAs detected in serum/plasma, CSF, and exosomes, stratified by clinical application. By mapping upregulated oncomiRs and downregulated tumor suppressors to diagnostic, prognostic, and monitoring contexts, it illustrates how liquid biopsy approaches may complement conventional imaging and histopathological evaluation.

DISCUSSION

Limitations

Despite the promising utility demonstrated by circulating miRNAs in GBM monitoring, several critical limitations temper their clinical integration.

First, significant pre-analytical and technical inconsistencies exist across studies. Variation in sample types (serum versus plasma versus CSF), collection methods, and RNA extraction protocols leads to inconsistent results, and normalization strategies lack consensus, as endogenous controls may themselves be altered in disease states.[132]

Second, the biological complexity and origin ambiguity of circulating miRNAs represent a major challenge. These molecules can originate not only from tumor cells but also from reactive stromal or immune cells, especially via EVs. Although EVs offer a protected route for miRNA dissemination, their isolation may co-purify contaminants, and their cargo heterogeneity reflects diverse cellular processes rather than tumor biology alone.[133] This source-specificity issue is particularly acute in GBM, where circulating miRNAs reflect contributions from at least four distinct compartments: tumor cells; the tumor microenvironment (reactive astrocytes, microglia/macrophages, endothelial cells); peripheral immune cells, whose miRNA repertoires shift with systemic inflammation and treatment[134]; and platelets, which release abundant miRNAs upon activation and during serum coagulation. Because mature blood cells exhibit highly abundant and lineage-specific miRNA profiles, even minor hemolysis or incomplete platelet depletion during sample processing can mask or distort tumor-derived signals, and quality-control panels have been proposed to flag such contamination in clinical samples.[135] This is particularly relevant for candidate biomarkers such as miR-223, which is enriched in granulocytes and platelets and may fluctuate with treatment-associated changes in blood cell counts independently of tumor biology. The BBB adds a further layer of complexity: in GBM, BBB disruption is heterogeneous in time and space and is likely to modulate the passage of tumor-derived miRNAs into the systemic circulation, contributing to inter-study variability and reinforcing the appeal of CSF as a tumor-proximal compartment less affected by these confounders. Emerging strategies to mitigate these limitations include surface-marker-based enrichment of tumor-derived EVs and focused ultrasound-induced BBB opening to facilitate the release of brain tumor biomarkers into the bloodstream.[136]

Third, many studies to date are based on small, single-institution cohorts, which may not reflect broader patient diversity or capture longitudinal dynamics. The absence of large-scale prospective validation significantly diminishes the generalizability of identified miRNA signatures.[133]

Fourth, analytical platform constraints further complicate clinical translation. As noted by Godoy et al., RT-qPCR arrays show high sensitivity for known targets and good reproducibility, but microarrays result in higher false-positive rates and correlate poorly with qPCR, while RNA-seq remains costly and suffers from standardization challenges.[137] Additionally, electrochemical miRNA biosensors provide rapid and target-specific detection at lower cost, yet their clinical translation remains limited by issues of reproducibility, signal specificity, and cost-effectiveness.[138]

Finally, no circulating miRNA has been validated head to head against MRI or established molecular markers such as MGMT methylation in randomized clinical trials. Regulatory acceptance will require evidence of improved clinical decision-making through prospective, multicenter studies.[133]

Future perspectives

Several avenues hold promise for advancing circulating miRNAs toward clinical implementation. On the technological front, novel multiplexed biosensors leveraging CRISPR-Cas systems, nanostructured probes, and microfluidic platforms are emerging as highly sensitive, rapid, and low-cost approaches for circulating miRNA detection, with potential for point-of-care GBM monitoring applications.[139,140]

The integration of miRNA profiles with other liquid biopsy analytes (e.g., ctDNA), proteomic markers, imaging features, and clinical variables—analyzed via machine-learning algorithms—could yield robust, dynamic biomarker signatures that better stratify patients and guide therapeutic decisions.[141] In parallel, exosome cargo holds dual potential: as stable carriers of tumor-specific miRNAs for diagnostic applications, and as natural vehicles for delivering therapeutic oligonucleotides across the BBB, thereby potentially overcoming a major delivery challenge in GBM treatment.[133,142]

To bridge bench-to-bedside translation, standardized protocols for sample handling, miRNA quantification, and data analysis must be established. Large-scale, multicenter trial designs incorporating miRNA endpoints alongside radiographic and molecular metrics will be essential to confirm clinical utility and achieve regulatory acceptance.

In summary, while circulating miRNAs offer a compelling path toward non-invasive, dynamic GBM monitoring, technical, biological, and clinical hurdles must be addressed. Advances in detection technology, multi-omic integration, and rigorous validation frameworks represent the most promising avenues to realize their full translational potential—moving toward personalized, real-time monitoring in neuro-oncology.

Conclusion

Circulating miRNAs represent highly promising biomarkers for glioma management, with evidence supporting their role across diagnosis, prognosis, treatment monitoring, and recurrence assessment. However, significant challenges remain, particularly regarding technical standardization and prospective validation. Integration with multi-omic platforms, advanced detection technologies, and clinical-trial frameworks may ultimately pave the way toward their adoption in precision neuro-oncology.

Table 5. Circulating miRNAs for monitoring treatment response in glioma

miR	Fluid/matrix	Expression change vs. treatment course	Treatment type	Clinical note	Authors (name, year)
miR-10b	CSF/serum	↑ associated with progression; decrease post-response	RT + TMZ; general follow-up	stemness/invasion miRNA; higher levels track active disease	Kopková, 2019[41]
miR-21	serum/plasma/CSF exosomes	↓ in responders; ↑/remain high in non-responders; ↑ at progression	Stupp protocol (RT + TMZ); Anti-angiogenic therapy (bevacizumab); general longitudinal follow-up	tracks disease activity; dynamic across therapy; best-validated liquid marker	Hartanto, 2024[125]; Siegal, 2016[126]; Olioso, 2021[102]; Wang, 2012[83]
miR-124-3p	serum exosomes	↑ in progressive disease; normalization suggests response	general longitudinal follow-up	tumor suppressor in tissue; exosomal release increases with progression	Olioso, 2021[102]
miR-128	plasma/serum	candidate: ↓ with progression; ↑ with disease control (cohort-dependent)	RT + TMZ; general follow-up	neuronal identity; tissue suppressor—circulating dynamics reported in your draft	Wang, 2012[83]
miR-155	plasma/serum	dynamic across treatment; ↑ may herald progression; ↓ with response	RT + TMZ; general longitudinal follow-up	inflammation/immune-related miRNA; included in longitudinal panels	Wu, 2022[97]
miR-181d	plasma/serum; Exosomes	higher baseline → better response; low/declining levels → poor response	RT + TMZ (MGMT-linked)	predictive of MGMT status and treatment response; useful for monitoring	Sippl, 2022[95]; Stakaitis, 2020[96]
miR-185	plasma	reported ↓ after therapy in responders (normalize with disease control)	surgery + adjuvant therapy; RT + TMZ	candidate longitudinal marker; needs standardized matrices	Tang, 2015[121]
miR-210	serum/Exosomes	↓ with response (reduced hypoxia); ↑ with resistance/ progression	RT + TMZ; bevacizumab (anti-angiogenic)	hypoxia-related oncomiR; reflects tumor oxygenatioN/ Angiogenesis	Lan, 2020[101]
miR-222	serum/CSF; EVs	↓ in responders; ↑ in non-responders; rises with progression	Stupp protocol (RT + TMZ); anti-angiogenic therapy (bevacizumab)	parallels miR-21 dynamics; linked to DNA repair and drug resistance	Hartanto, 2024[125]; Siegal, 2016; Olioso, 2021[102]
miR-342-3p	plasma	candidate: changes reported with treatment course	RT + TMZ; general follow-up	signal noted alongside miR-128 in responders vs. non-responders	Wang, 2012[83]
miR-410	plasma	dynamic across treatment; trends with response (panel data)	RT + TMZ	included with miR-155/181 family in longitudinal assessments	Wu, 2022[97] (panel)
miR-485-3p	serum	returns toward normal after surgery/therapy in responders	surgery + adjuvant therapy	low levels associate with poorer outcome; dynamic normalization post-treatment	Wang, 2017[108]
miR-1238	serum exosomes	↑ in non-responders/TMZ-resistant; decreases with restored sensitivity	TMZ	exosomal transfer mediates chemoresistance; candidate for real-time resistance tracking	Yin, 2019[127]

Dynamic changes in miRNA expression are reported across surgical, chemoradiation, and anti-angiogenic therapy contexts. CSF, cerebrospinal fluid; EV, extracellular vesicle; MGMT, O6-methylguanine-DNA methyltransferase; miR, microRNA; RT, radiotherapy; TMZ, temozolomide.

Table 6. Circulating miRNAs for differentiating true tumor recurrence from pseudo-progression[128]

miR	Fluid/matrix	Expression dynamics	Tumor type/context	Clinical note	Authors (name, year)
miR-223	serum	↓ after resection; ↑ at recurrence	LGG	dynamic correlation with tumor volume; did not increase in pseudo-progression	Morokoff, 2020[128]
miR-320e	serum	↓ after resection; ↑ at recurrence	GBM	correlates with tumor volume; stable in pseudo-progression	Morokoff, 2020[128]
miR-21	serum	contributes to diagnostic 4-miR panel; dynamic levels reported	GBM/LGG	combined with miR-223, miR-320e, and miR-23a improves diagnostic accuracy	Morokoff, 2020[128]
miR-23a	serum	part of diagnostic 4-miR panel	GBM/LGG	less dynamic individually, but increases accuracy when combined	Morokoff, 2020[128]

Serum miRNA dynamics were assessed longitudinally in glioma patients with serial sampling. GBM, glioblastoma; LGG, low-grade glioma; miR, microRNA.

Materials and methods

Search strategy

This review comprises two parts: a narrative overview of circulating miRNA biology, pre-analytical handling, and detection methods; and a systematic review conducted according to PRISMA guidelines to identify clinical studies on circulating miRNAs as biomarkers in glioma.

Literature searches were performed in PubMed, Embase, Scopus, and Web of Science using terms combining "glioma" or "glioblastoma" with "microRNA," "miRNA," and "circulating." Boolean operators and truncation were applied. The search was limited to peer-reviewed articles published in English from January 2000 to August 2025. Additional records were identified from reference lists of selected studies. All references were imported into a citation manager and duplicates removed.

Inclusion and exclusion criteria

Studies were included if they (1) reported original data on circulating miRNAs in glioma patients; (2) used blood (serum, plasma) or CSF as the miRNA source; (3) reported diagnostic, prognostic, predictive, or monitoring outcomes; and (4) were published in peer-reviewed English-language journals. Studies based exclusively on tissue miRNAs, non-original works (reviews, editorials, case reports, conference abstracts), studies without a clinical cohort, and those with insufficient methodological detail were excluded.

Study selection and data extraction

Titles and abstracts were independently reviewed by two investigators. Full texts of potentially relevant articles were retrieved for detailed evaluation; disagreements were resolved by consensus or consultation with a third reviewer. Data were extracted using a standardized form covering study characteristics (first author, year, country, design, sample size), patient characteristics (age, sex, histological and molecular subtype, treatment context), biofluid source, miRNA(s) analyzed, detection method, normalization strategy, and clinical outcomes.

Due to heterogeneity in study design, patient cohorts, biofluid sources, and outcome measures, a quantitative meta-analysis was not feasible. Findings were therefore synthesized narratively, emphasizing recurring miRNA candidates, study quality, and consistency

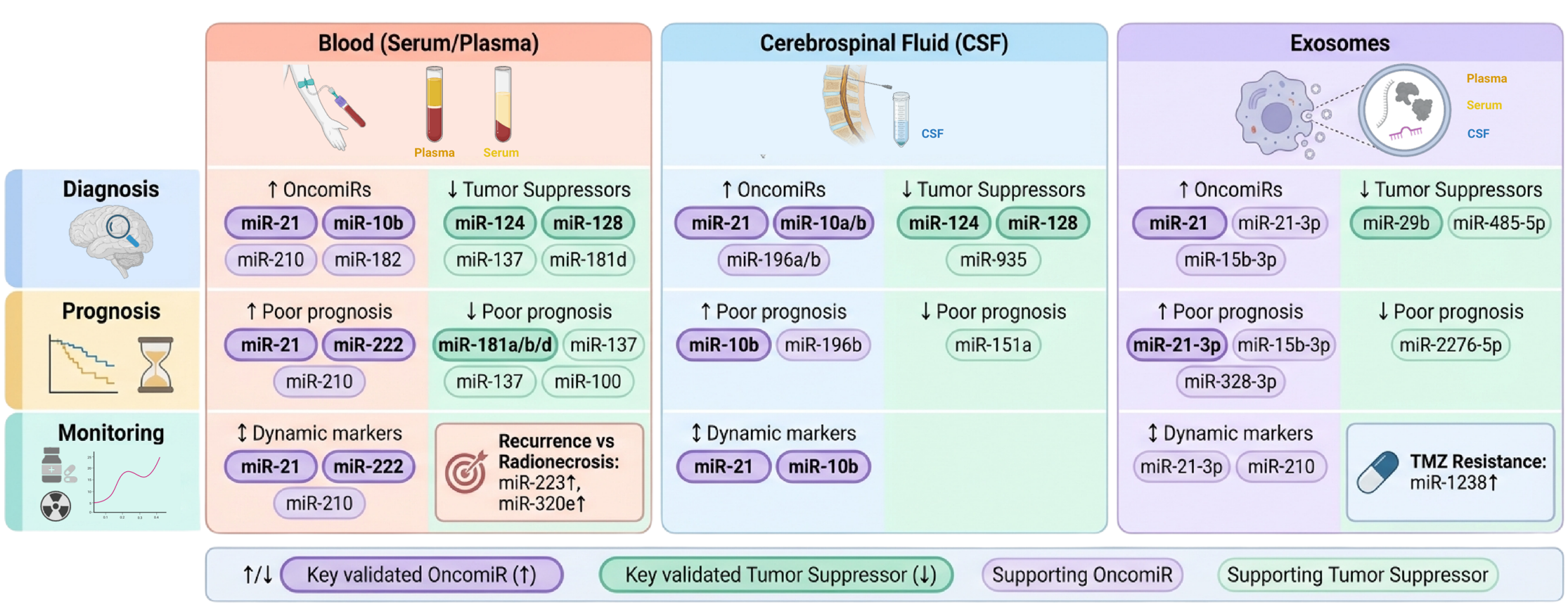

Figure 1. Integrative overview of circulating miRNAs detected in serum/plasma, CSF, and exosomes, stratified by clinical application (diagnosis, prognosis, and monitoring)

OncomiRs are shown in red (upregulated); tumor-suppressor miRNAs are shown in blue (downregulated).

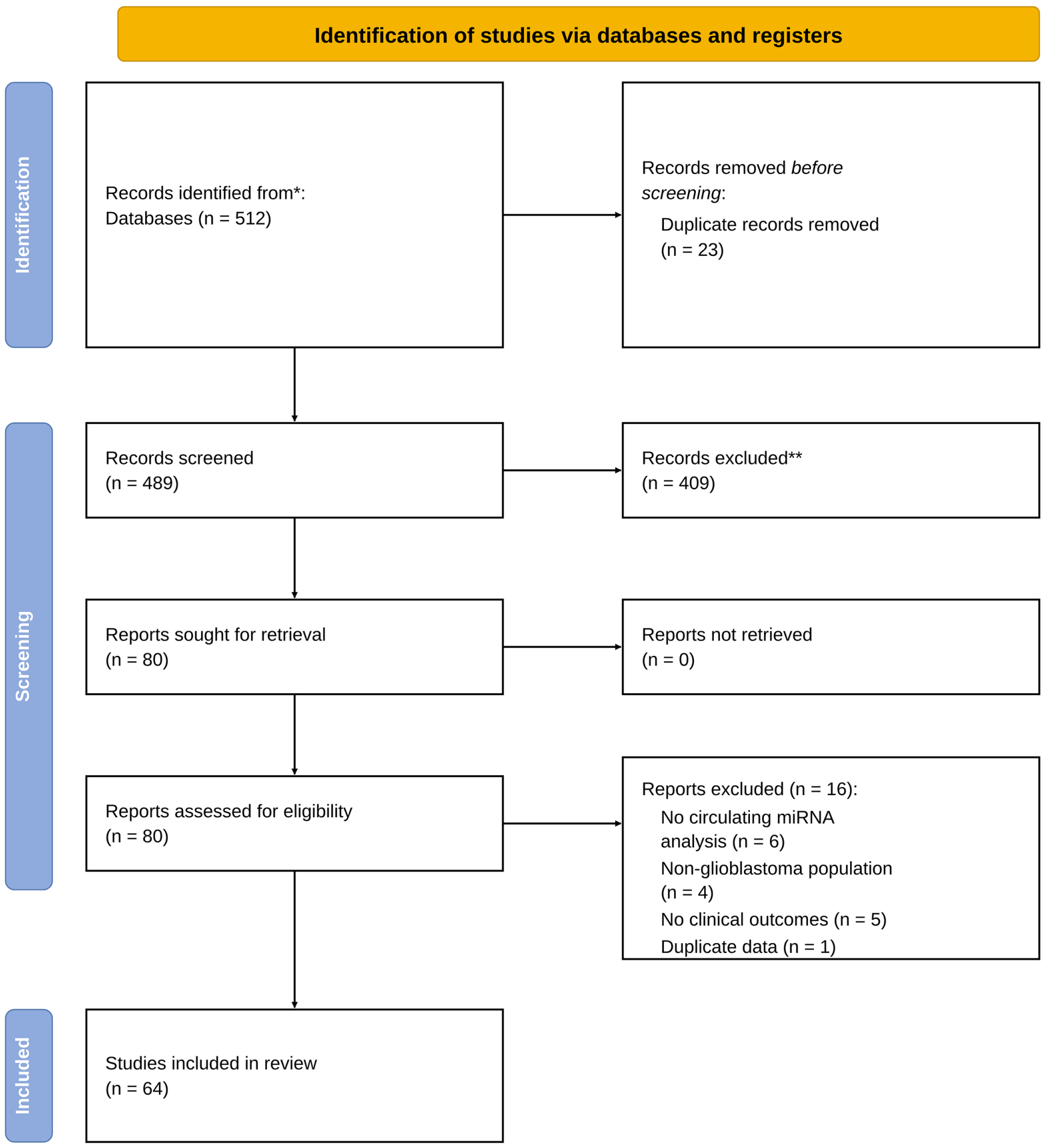

Figure 2. PRISMA flow diagram of the systematic literature search and study selection process.

of results. The initial PubMed search yielded 512 records. After duplicate removal, 489 records were screened, of which 409 were excluded. Eighty full-text articles were assessed; 16 were excluded (six without circulating miRNA analysis, four including non-glioma populations, five lacking clinical outcomes, and one duplicate dataset). Sixty-four studies met the inclusion criteria (Figure 2).

Study quality assessment

Given the marked heterogeneity of available studies and the absence of a single validated risk-of-bias tool tailored to circulating miRNA biomarker research, a pragmatic qualitative grading framework was applied to characterize the methodological strength of each included study rather than to formally pool or exclude studies. Five complementary dimensions were considered: (1) cohort size, stratified as small (<50 patients), moderate (50–100), or large (>100); (2) study design, distinguishing prospective from retrospective enrollment; (3) inclusion of an independent validation cohort or external dataset; (4) control of pre-analytical and technical variables, including hemolysis assessment, normalization strategy, and standardized RNA extraction protocols; and (5) statistical rigor, with multivariable analyses controlling for known prognostic factors (age, KPS, IDH/MGMT status, treatment) considered superior to univariate analyses alone.

These criteria informed the qualitative weighting of evidence throughout the present review and underlie the consistency tiers reported in Table S1. Particular emphasis was placed on candidate miRNAs whose performance was replicated across independent cohorts, biofluid types, and detection platforms. Conversely, results derived exclusively from small, single-institution, retrospective cohorts without independent validation are flagged in the narrative as preliminary and requiring confirmation in larger prospective studies. This framework should be regarded as an informal and pragmatic appraisal rather than as a formal risk-of-bias assessment such as ROBINS-I or QUADAS-2, which were not directly applicable given the predominantly observational and biomarker-discovery nature of the included studies.

DATA AND CODE AVAILABILITY

This study did not generate new data or code. All data discussed in this review were obtained from previously published studies, which are cited in the reference list and are publicly available through their original publications.

ACKNOWLEDGMENTS

The authors thank the medical and technical staff at CHU Caen and GIP CYCERON for their support in neuro-oncological research. This work was supported by the Région Normandie (GliomiR, 2023-A01732-43).

AUTHOR CONTRIBUTIONS

A.L., conceptualization, writing – original draft, and writing – review and editing; E.E., writing – review and editing; G.L., supervision and writing – review and editing.

DECLARATION OF INTERESTS

The authors declare no competing interests.

SUPPLEMENTAL INFORMATION

Supplemental information can be found online at https://doi.org/10.1016/j.omton.2026.201308.

REFERENCES

1. Kros, J.M., Mustafa, D.M., Dekker, L.J.M., Sillevis Smitt, P.A.E., Luider, T.M., and Zheng, P.P. (2015). Circulating glioma biomarkers. Neuro Oncol. *17*, 343–360. https://doi.org/10.1093/neuonc/nou207.

2. Pope, W.B., and Brandal, G. (2018). Conventional and advanced magnetic resonance imaging in patients with high-grade glioma. Q. J. Nucl. Med. Mol. Imaging *62*, 239–253. https://doi.org/10.23736/S1824-4785.18.03086-8.

3. Kreth, S., Thon, N., and Kreth, F.W. (2014). Epigenetics in human gliomas. Cancer Lett. *342*, 185–192. https://doi.org/10.1016/j.canlet.2012.04.008.

4. Ma, C., Nguyen, H.P.T., Luwor, R.B., Stylli, S.S., Gogos, A., Paradiso, L., Kaye, A.H., and Morokoff, A.P. (2018). A comprehensive meta-analysis of circulation miRNAs in glioma as potential diagnostic biomarker. PLoS One *13*, e0189452. https://doi.org/10.1371/journal.pone.0189452.

5. Singh, G., Manjila, S., Sakla, N., True, A., Wardeh, A.H., Beig, N., Vaysberg, A., Matthews, J., Prasanna, P., and Spektor, V. (2021). Radiomics and radiogenomics in gliomas: a contemporary update. Br. J. Cancer *125*, 641–657. https://doi.org/10.1038/s41416-021-01387-w.

6. Mandel, P., and Métais, P. (1948). Les acides nucléiques du plasma sanguin chez l'homme. C. R. Seances Soc. Biol. Fil. *142*, 241–243.

7. Leon, S.A., Shapiro, B., Sklaroff, D.M., and Yaros, M.J. (1977). Free DNA in the serum of cancer patients and the effect of therapy. Cancer Res. *37*, 646–650.

8. Colmenares, R., Álvarez, N., Barrio, S., Martínez-López, J., and Ayala, R. (2022). The Minimal Residual Disease Using Liquid Biopsies in Hematological Malignancies. Cancers (Basel) *14*, 1310. https://doi.org/10.3390/cancers14051310.

9. Wan, J.C.M., Massie, C., Garcia-Corbacho, J., Mouliere, F., Brenton, J.D., Caldas, C., Pacey, S., Baird, R., and Rosenfeld, N. (2017). Liquid biopsies come of age: towards implementation of circulating tumour DNA. Nat. Rev. Cancer *17*, 223–238. https://doi.org/10.1038/nrc.2017.7.

10. Xu, X., Iqbal, Z., Xu, L., Wen, C., Duan, L., Xia, J., Yang, N., Zhang, Y., and Liang, Y. (2024). Brain-derived extracellular vesicles: Potential diagnostic biomarkers for central nervous system diseases. Psychiatry Clin. Neurosci. *78*, 83–96. https://doi.org/10.1111/pcn.13610.

11. Cheng, G. (2015). Circulating miRNAs: roles in cancer diagnosis, prognosis and therapy. Adv. Drug Deliv. Rev. *81*, 75–93. https://doi.org/10.1016/j.addr.2014.09.001.

12. Ray, A., and Vohra, T.K. (2022). Liquid biopsy-from bench to bedside. Neurooncol. Adv. *4*, ii66–ii72. https://doi.org/10.1093/noajnl/vdac037.

13. Marcuello, M., Vymetalkova, V., Neves, R.P.L., Duran-Sanchon, S., Vedeld, H.M., Tham, E., van Dalum, G., Flügen, G., Garcia-Barberan, V., Fijneman, R.J., et al. (2019). Circulating biomarkers for early detection and clinical management of colorectal cancer. Mol. Aspects Med. *69*, 107–122. https://doi.org/10.1016/j.mam.2019.06.002.

14. Zhang, Y., Dutta, A., and Abounader, R. (2012). The role of microRNAs in glioma initiation and progression. Front. Biosci. *17*, 700–712. https://doi.org/10.2741/3952.

15. Bartel, D.P. (2004). MicroRNAs: genomics, biogenesis, mechanism, and function. Cell *116*, 281–297. https://doi.org/10.1016/s0092-8674(04)00045-5.

16. O'Brien, J., Hayder, H., Zayed, Y., and Peng, C. (2018). Overview of MicroRNA Biogenesis, Mechanisms of Actions, and Circulation. Front. Endocrinol. *9*, 402. https://doi.org/10.3389/fendo.2018.00402.

17. Shang, R., Lee, S., Senavirathne, G., and Lai, E.C. (2023). microRNAs in action: biogenesis, function and regulation. Nat. Rev. Genet. *24*, 816–833. https://doi.org/10.1038/s41576-023-00611-y.

18. Kim, H., Lee, Y.Y., and Kim, V.N. (2025). The biogenesis and regulation of animal microRNAs. Nat. Rev. Mol. Cell Biol. *26*, 276–296. https://doi.org/10.1038/s41580-024-00805-0.

19. Calin, G.A., Dumitru, C.D., Shimizu, M., Bichi, R., Zupo, S., Noch, E., Aldler, H., Rattan, S., Keating, M., Rai, K., et al. (2002). Frequent deletions and down-regulation of micro-RNA genes miR15 and miR16 at 13q14 in chronic lymphocytic leukemia. Proc. Natl. Acad. Sci. USA *99*, 15524–15529. https://doi.org/10.1073/pnas.242606799.

20. Lu, J., Getz, G., Miska, E.A., Alvarez-Saavedra, E., Lamb, J., Peck, D., Sweet-Cordero, A., Ebert, B.L., Mak, R.H., Ferrando, A.A., et al. (2005). MicroRNA expression profiles classify human cancers. Nature *435*, 834–838. https://doi.org/10.1038/nature03702.

21. Esquela-Kerscher, A., and Slack, F.J. (2006). Oncomirs - microRNAs with a role in cancer. Nat. Rev. Cancer *6*, 259–269. https://doi.org/10.1038/nrc1840.

22. Zhao, W., Gupta, A., Krawczyk, J., and Gupta, S. (2022). The miR-17-92 cluster: Yin and Yang in human cancers. Cancer Treat. Res. Commun. *33*, 100647. https://doi.org/10.1016/j.ctarc.2022.100647.

23. Syed, R.U., Banu, H., Alshammrani, A., Alshammari, M.D., G, S.K., Kadimpati, K.K., Khalifa, A.A.S., Aboshouk, N.A.M., Almarir, A.M., Hussain, A., and Alahmed, F.K. (2024). MicroRNA-21 (miR-21) in breast cancer: From apoptosis dysregulation to therapeutic opportunities. Pathol. Res. Pract. *262*, 155572. https://doi.org/10.1016/j.prp.2024.155572.

24. Mitchell, P.S., Parkin, R.K., Kroh, E.M., Fritz, B.R., Wyman, S.K., Pogosova-Agadjanyan, E.L., Peterson, A., Noteboom, J., O'Briant, K.C., Allen, A., et al. (2008). Circulating microRNAs as stable blood-based markers for cancer detection. Proc. Natl. Acad. Sci. USA *105*, 10513–10518. https://doi.org/10.1073/pnas.0804549105.

25. Diener, C., Keller, A., and Meese, E. (2022). Emerging concepts of miRNA therapeutics: from cells to clinic. Trends Genet. *38*, 613–626. https://doi.org/10.1016/j.tig.2022.02.006.

26. Sufianov, A., Begliarzade, S., Ilyasova, T., Liang, Y., and Beylerli, O. (2022). MicroRNAs as prognostic markers and therapeutic targets in gliomas. Noncoding. RNA Res. *7*, 171–177. https://doi.org/10.1016/j.ncrna.2022.07.001.

27. Iguchi, H., Kosaka, N., and Ochiya, T. (2010). Versatile applications of microRNA in anti-cancer drug discovery: from therapeutics to biomarkers. Curr. Drug Discov. Technol. *7*, 95–105. https://doi.org/10.2174/157016310793180648.

28. Banelli, B., Forlani, A., Allemanni, G., Morabito, A., Pistillo, M.P., and Romani, M. (2017). MicroRNA in Glioblastoma: An Overview. Int. J. Genomics *2017*, 7639084. https://doi.org/10.1155/2017/7639084.

29. Farsetti, A., Illi, B., and Gaetano, C. (2023). How epigenetics impacts on human diseases. Eur. J. Intern. Med. *114*, 15–22. https://doi.org/10.1016/j.ejim.2023.05.036.

30. Liu, Z., Jiang, Z., Huang, J., Huang, S., Li, Y., Yu, S., Yu, S., and Liu, X. (2014). miR-7 inhibits glioblastoma growth by simultaneously interfering with the PI3K/ATK and Raf/MEK/ERK pathways. Int. J. Oncol. *44*, 1571–1580. https://doi.org/10.3892/ijo.2014.2322.

31. Zhang, Y., Chao, T., Li, R., Liu, W., Chen, Y., Yan, X., Gong, Y., Yin, B., Liu, W., Qiang, B., et al. (2009). MicroRNA-128 inhibits glioma cells proliferation by targeting transcription factor E2F3a. J. Mol. Med. *87*, 43–51. https://doi.org/10.1007/s00109-008-0403-6.

32. Silber, J., Lim, D.A., Petritsch, C., Persson, A.I., Maunakea, A.K., Yu, M., Vandenberg, S.R., Ginzinger, D.G., James, C.D., Costello, J.F., et al. (2008). miR-124 and miR-137 inhibit proliferation of glioblastoma multiforme cells and induce differentiation of brain tumor stem cells. BMC Med. *6*, 14. https://doi.org/10.1186/1741-7015-6-14.

33. Rahmani, F., Hashemian, P., Tabrizi, A.T., Ghorbani, Z., Ziaeemehr, A., Alijannejad, S., Ferns, G.A., Avan, A., and Shahidsales, S. (2023). Regulatory role of miRNAs on Wnt/β-catenin signaling in tumorigenesis of glioblastoma. Indian J. Cancer *60*, 295–302. https://doi.org/10.4103/ijc.IJC_251_21.

34. Chan, J.A., Krichevsky, A.M., and Kosik, K.S. (2005). MicroRNA-21 is an antiapoptotic factor in human glioblastoma cells. Cancer Res. *65*, 6029–6033. https://doi.org/10.1158/0008-5472.CAN-05-0137.

35. Kwak, H.J., Kim, Y.J., Chun, K.R., Woo, Y.M., Park, S.J., Jeong, J.A., Jo, S.H., Kim, T.H., Min, H.S., Chae, J.S., et al. (2011). Downregulation of Spry2 by miR-21 triggers malignancy in human gliomas. Oncogene *30*, 2433–2442. https://doi.org/10.1038/onc.2010.620.

36. Chen, M., Medarova, Z., and Moore, A. (2021). Role of microRNAs in glioblastoma. Oncotarget *12*, 1707–1723. https://doi.org/10.18632/oncotarget.28039.

37. Gabriely, G., Yi, M., Narayan, R.S., Niers, J.M., Wurdinger, T., Imitola, J., Ligon, K.L., Kesari, S., Esau, C., Stephens, R.M., et al. (2011). Human glioma growth is controlled by microRNA-10b. Cancer Res. *71*, 3563–3572. https://doi.org/10.1158/0008-5472.CAN-10-3568.

38. Fang, L., Deng, Z., Shatseva, T., Yang, J., Peng, C., Du, W.W., Yee, A.J., Ang, L.C., He, C., Shan, S.W., and Yang, B.B. (2011). MicroRNA miR-93 promotes tumor growth and angiogenesis by targeting integrin-β8. Oncogene *30*, 806–821. https://doi.org/10.1038/onc.2010.465.

39. Makowska, M., Smolarz, B., and Romanowicz, H. (2023). microRNAs (miRNAs) in Glioblastoma Multiforme (GBM)-Recent Literature Review. Int. J. Mol. Sci. *24*, 3521. https://doi.org/10.3390/ijms24043521.

40. Ordóñez-Rubiano, E.G., Rincón-Arias, N., Espinosa, S., Shelton, W.J., Salazar, A.F., Cómbita, A., Baldoncini, M., Luzzi, S., Payán-Gómez, C., Gómez- Amarillo, D.F., et al. (2024). The potential of miRNA-based approaches in glioblastoma: An update in current advances and future perspectives. Curr. Res. Pharmacol. Drug Discov. *7*, 100193. https://doi.org/10.1016/j.crphar.2024.100193.

41. Kopkova, A., Sana, J., Machackova, T., Vecera, M., Radova, L., Trachtova, K., Vybihal, V., Smrcka, M., Kazda, T., Slaby, O., and Fadrus, P. (2019). Cerebrospinal Fluid MicroRNA Signatures as Diagnostic Biomarkers in Brain Tumors. Cancers *11*, 1546. https://doi.org/10.3390/cancers11101546.

42. Sasayama, T., Tanaka, K., and Kohmura, E. (2016). The Roles of MicroRNAs in Glioblastoma Biology and Biomarker. In Neurooncology - Newer Developments. https://doi.org/10.5772/63243.

43. Cunha e Rocha, K., Ying, W., and Olefsky, J.M. (2024). Exosome-Mediated Impact on Systemic Metabolism. Annu. Rev. Physiol. *86*, 225–253. https://doi.org/10.1146/annurev-physiol-042222-024535.

44. Cui, M., Wang, H., Yao, X., Zhang, D., Xie, Y., Cui, R., and Zhang, X. (2019). Circulating MicroRNAs in Cancer: Potential and Challenge. Front. Genet. *10*, 626. https://doi.org/10.3389/fgene.2019.00626.

45. Simionescu, N., Zonda, R., Petrovici, A.R., and Georgescu, A. (2021). The Multifaceted Role of Extracellular Vesicles in Glioblastoma: microRNA Nanocarriers for Disease Progression and Gene Therapy. Pharmaceutics *13*, 988. https://doi.org/10.3390/pharmaceutics13070988.

46. Arroyo, J.D., Chevillet, J.R., Kroh, E.M., Ruf, I.K., Pritchard, C.C., Gibson, D.F., Mitchell, P.S., Bennett, C.F., Pogosova-Agadjanyan, E.L., Stirewalt, D.L., et al. (2011). Argonaute2 complexes carry a population of circulating microRNAs independent of vesicles in human plasma. Proc. Natl. Acad. Sci. USA *108*, 5003–5008. https://doi.org/10.1073/pnas.1019055108.

47. Wang, K., Yuan, Y., Cho, J.H., McClarty, S., Baxter, D., and Galas, D.J. (2012). Comparing the MicroRNA Spectrum between Serum and Plasma. PLoS One *7*, e41561. https://doi.org/10.1371/journal.pone.0041561.

48. Sandau, U.S., Wiedrick, J.T., McFarland, T.J., Galasko, D.R., Fanning, Z., Quinn, J.F., and Saugstad, J.A. (2024). Analysis of the longitudinal stability of human plasma miRNAs and implications for disease biomarkers. Sci. Rep. *14*, 2148. https://doi.org/10.1038/s41598-024-52681-5.

49. Dufourd, T., Robil, N., Mallet, D., Carcenac, C., Boulet, S., Brishoual, S., Rabois, E., Houeto, J.L., de la Grange, P., and Carnicella, S. (2019). Plasma or serum?.A qualitative study on rodents and humans using high-throughput microRNA sequencing for circulating biomarkers. Biol. Methods Protoc. *4*, bpz006. https://doi.org/10.1093/biomethods/bpz006.

50. Burgos, K.L., Javaherian, A., Bomprezzi, R., Ghaffari, L., Rhodes, S., Courtright, A., Tembe, W., Kim, S., Metpally, R., and Van Keuren-Jensen, K. (2013). Identification of extracellular miRNA in human cerebrospinal fluid by next-generation sequencing. RNA *19*, 712–722. https://doi.org/10.1261/rna.036863.112.

51. Godoy, P.M., Bhakta, N.R., Barczak, A.J., Cakmak, H., Fisher, S., MacKenzie, T.C., Patel, T., Price, R.W., Smith, J.F., Woodruff, P.G., and Erle, D.J. (2018). Large Differences in Small RNA Composition Between Human Biofluids. Cell Rep. *25*, 1346–1358. https://doi.org/10.1016/j.celrep.2018.10.014.

52. Alsop, E., Meechoovet, B., Kitchen, R., Sweeney, T., Beach, T.G., Serrano, G.E., Hutchins, E., Ghiran, I., Reiman, R., Syring, M., et al. (2022). A Novel Tissue Atlas and Online Tool for the Interrogation of Small RNA Expression in Human Tissues and Biofluids. Front. Cell Dev. Biol. *10*, 804164. https://doi.org/10.3389/fcell.2022.804164.

53. Max, K.E.A., Bertram, K., Akat, K.M., Bogardus, K.A., Li, J., Morozov, P., Ben-Dov, I.Z., Li, X., Weiss, Z.R., Azizian, A., et al. (2018). Human plasma and serum extracellular small RNA reference profiles and their clinical utility. Proc. Natl. Acad. Sci. USA *115*, E5334–E5343. https://doi.org/10.1073/pnas.1714397115.

54. Freedman, J.E., Gerstein, M., Mick, E., Rozowsky, J., Levy, D., Kitchen, R., Das, S., Shah, R., Danielson, K., Beaulieu, L., et al. (2016). Diverse human extracellular RNAs are widely detected in human plasma. Nat. Commun. *7*, 11106.

55. Srinivasan, S., Yeri, A., Cheah, P.S., Chung, A., Danielson, K., De Hoff, P., Filant, J., Laurent, C.D., Laurent, L.D., Magee, R., et al. (2019). Small RNA Sequencing across Diverse Biofluids Identifies Optimal Methods for exRNA Isolation. Cell *177*, 446–462.e16. https://doi.org/10.1016/j.cell.2019.03.024.

56. Murillo, O.D., Thistlethwaite, W., Rozowsky, J., Subramanian, S.L., Lucero, R., Shah, N., Jackson, A.R., Srinivasan, S., Chung, A., Laurent, C.D., et al. (2019). exRNA Atlas Analysis Reveals Distinct Extracellular RNA Cargo Types and Their Carriers Present across Human Biofluids. Cell *177*, 463–477.e15. https://doi.org/10.1016/j.cell.2019.02.018.

57. Michel, U., Shomroni, O., Müller, B., Lange, P., Salinas, G., Bähr, M., and Koch, J.C. (2022). Small and long RNA transcriptome of whole human cerebrospinal fluid and serum as compared to their extracellular vesicle fractions reveal profound differences in expression patterns and impacts on biological processes. J. Transl. Med. *20*, 413. https://doi.org/10.1186/s12967-022-03612-3.

58. Weber, J.A., Baxter, D.H., Zhang, S., Huang, D.Y., Huang, K.H., Lee, M.J., Galas, D.J., and Wang, K. (2010). The microRNA spectrum in 12 body fluids. Clin. Chem. *56*, 1733–1741. https://doi.org/10.1373/clinchem.2010.147405.

59. Zhao, H., Shen, J., Hu, Q., Davis, W., Medico, L., Wang, D., Yan, L., Guo, Y., Liu, B., Qin, M., et al. (2014). Effects of preanalytic variables on circulating microRNAs in whole blood. Cancer Epidemiol. Biomarkers Prev. *23*, 2643–2648. https://doi.org/10.1158/1055-9965.EPI-14-0550.

60. Jarry, J., Schadendorf, D., Greenwood, C., Spatz, A., and van Kempen, L.C. (2014). The validity of circulating microRNAs in oncology: five years of challenges and contradictions. Mol. Oncol. *8*, 819–829. https://doi.org/10.1016/j.molonc.2014.02.009.

61. Skouras, P., Markouli, M., Kalamatianos, T., Stranjalis, G., Korkolopoulou, P., and Piperi, C. (2023). Advances on Liquid Biopsy Analysis for Glioma Diagnosis. Biomedicines *11*, 2371. https://doi.org/10.3390/biomedicines11092371.

62. Matias-Garcia, P.R., Wilson, R., Mussack, V., Reischl, E., Waldenberger, M., Gieger, C., Anton, G., Peters, A., and Kuehn-Steven, A. (2020). Impact of long-term storage and freeze-thawing on eight circulating microRNAs in plasma samples. PLoS One *15*, e0227648. https://doi.org/10.1371/journal.pone.0227648.

63. Seong, E., and George, N.M. (2020). Abstract 4831: Stability of draw-time microRNA concentration in blood collection tubes. Cancer Res. *80*, 4831. https://doi.org/10.1158/1538-7445.AM2020-4831.

64. Tiberio, P., Callari, M., Angeloni, V., Daidone, M.G., and Appierto, V. (2015). Challenges in using circulating miRNAs as cancer biomarkers. Biomed Res. Int. *2015*, 731479. https://doi.org/10.1155/2015/731479.

65. Kroh, E.M., Parkin, R.K., Mitchell, P.S., and Tewari, M. (2010). Analysis of circulating microRNA biomarkers in plasma and serum using quantitative reverse transcription-PCR (qRT-PCR). Methods *50*, 298–301. https://doi.org/10.1016/j.ymeth.2010.01.032.

66. Chen, C., Ridzon, D.A., Broomer, A.J., Zhou, Z., Lee, D.H., Nguyen, J.T., Barbisin, M., Xu, N.L., Mahuvakar, V.R., Andersen, M.R., et al. (2005). Real-time quantification of microRNAs by stem-loop RT-PCR. Nucleic Acids Res. *33*, e179. https://doi.org/10.1093/nar/gni178.

67. Dave, V.P., Ngo, T.A., Pernestig, A.K., Tilevik, D., Kant, K., Nguyen, T., Wolff, A., and Bang, D.D. (2019). MicroRNA amplification and detection technologies: opportunities and challenges for point of care diagnostics. Lab. Invest. *99*, 452–469. https://doi.org/10.1038/s41374-018-0143-3.

68. Love, C., and Dave, S. (2013). MicroRNA expression profiling using microarrays. Methods Mol. Biol. *999*, 285–296. https://doi.org/10.1007/978-1-62703-357-2_21.

69. Thomson, J.M., Parker, J.S., and Hammond, S.M. (2007). Microarray analysis of miRNA gene expression. Methods Enzymol. *427*, 107–122. https://doi.org/10.1016/S0076-6879(07)27006-5.

70. Williams, Z., Ben-Dov, I.Z., Elias, R., Mihailovic, A., Brown, M., Rosenwaks, Z., and Tuschl, T. (2013). Comprehensive profiling of circulating microRNA via small RNA sequencing of cDNA libraries reveals biomarker potential and limitations. Proc. Natl. Acad. Sci. USA *110*, 4255–4260. https://doi.org/10.1073/pnas.1214046110.

71. Giraldez, M.D., Spengler, R.M., Etheridge, A., Godoy, P.M., Barczak, A.J., Srinivasan, S., De Hoff, P.L., Tanriverdi, K., Courtright, A., Lu, S., et al. (2018). Comprehensive multi-center assessment of small RNA-seq methods for quantitative miRNA profiling. Nat. Biotechnol. *36*, 746–757. https://doi.org/10.1038/nbt.4183.

72. Dama, E., Colangelo, T., Cuttano, R., Dziadziuszko, R., Dandekar, T., Widlak, P., Rzyman, W., Veronesi, G., and Bianchi, F. (2025). A plasma 9-microRNA signature for lung cancer early detection: a multicenter analysis. Biomark. Res. *13*, 74. https://doi.org/10.1186/s40364-025-00787-x.

73. Ling, L., Aldoghachi, A.F., Chong, Z.X., Ho, W.Y., Yeap, S.K., Chin, R.J., Soo, E.Z.X., Khor, J.F., Yong, Y.L., Ling, J.L., et al. (2022). Addressing the Clinical Feasibility of Adopting Circulating miRNA for Breast Cancer Detection, Monitoring and Management with Artificial Intelligence and Machine Learning Platforms. Int. J. Mol. Sci. *23*, 15382. https://doi.org/10.3390/ijms232315382.

74. Broseghini, E., Fontana, B., Durante, G., Roncarati, R., and Ferracin, M. (2026). Quantification of Tissue and Circulating MicroRNAs by Droplet Digital PCR. Methods Mol. Biol. *2969*, 195–233. https://doi.org/10.1007/978-1-0716-4767-7_13.

75. Fattahi, M., Rahdan, F., Shaterabadi, D., Zamani Sani, M., Alizadeh, M., Khatami, S.H., Taheri-Anganeh, M., Movahedpour, A., and Ghasemi, H. (2024). MicroRNA biosensors for the detection of liver cancer. Clin. Chim. Acta *554*, 117796. https://doi.org/10.1016/j.cca.2024.117796.

76. Rahdan, F., Bina, F., Norouz Dolatabadi, E., Shaterabadi, D., Khatami, S.H., Karami, Y., Dorosti, N., Taheri-Anganeh, M., Asadi, P., Soltani, R., et al. (2023). MicroRNA electrochemical biosensors for pancreatic cancer. Clin. Chim. Acta *548*, 117472. https://doi.org/10.1016/j.cca.2023.117472.

77. Herman, A., Gruden, K., Blejec, A., Podpečan, V., Motaln, H., Rožman, P., Hren, M., Zupančič, K., Veber, M., Verbovšek, U., et al. (2015). Analysis of Glioblastoma Patients' Plasma Revealed the Presence of MicroRNAs with a Prognostic Impact on Survival and Those of Viral Origin. PLoS One *10*, e0125791.

78. Díaz Méndez, A.B., Sacconi, A., Tremante, E., Lulli, V., Caprara, V., Rosanò, L., Goeman, F., Carosi, M., Di Giuliani, M., Vari, G., et al. (2023). A diagnostic circulating miRNA signature as orchestrator of cell invasion via TKS4/TKS5/EFHD2 modulation in human gliomas. J. Exp. Clin. Cancer Res. *42*, 66. https://doi.org/10.1186/s13046-023-02639-8.

79. Teplyuk, N.M., Mollenhauer, B., Gabriely, G., Giese, A., Kim, E., Smolsky, M., Kim, R.Y., Saria, M.G., Pastorino, S., Kesari, S., and Krichevsky, A.M. (2012). MicroRNAs in cerebrospinal fluid identify glioblastoma and metastatic brain cancers and reflect disease activity. Neuro Oncol. *14*, 689–700. https://doi.org/10.1093/neuonc/nos074.

80. Tzaridis, T., Reiners, K.S., Weller, J., Bachurski, D., Schäfer, N., Schaub, C., Hallek, M., Scheffler, B., Glas, M., Herrlinger, U., et al. (2020). Analysis of Serum miRNA in Glioblastoma Patients: CD44-Based Enrichment of Extracellular Vesicles Enhances Specificity for the Prognostic Signature. Int. J. Mol. Sci. *21*, 7211. https://doi.org/10.3390/ijms21197211.

81. Akers, J.C., Ramakrishnan, V., Kim, R., Skog, J., Nakano, I., Pingle, S., Kalinina, J., Hua, W., Kesari, S., Mao, Y., et al. (2013). MiR-21 in the extracellular vesicles (EVs) of cerebrospinal fluid (CSF): a platform for glioblastoma biomarker development. PLoS One *8*, e78115. https://doi.org/10.1371/journal.pone.0078115.

82. Baraniskin, A., Kuhnhenn, J., Schlegel, U., Maghnouj, A., Zöllner, H., Schmiegel, W., Hahn, S., and Schroers, R. (2012). Identification of microRNAs in the cerebrospinal fluid as biomarker for the diagnosis of glioma. Neuro Oncol. *14*, 29–33. https://doi.org/10.1093/neuonc/nor169.

83. Wang, Q., Li, P., Li, A., Jiang, W., Wang, H., Wang, J., and Xie, K. (2012). Plasma specific miRNAs as predictive biomarkers for diagnosis and prognosis of glioma. J. Exp. Clin. Cancer Res. *31*, 97. https://doi.org/10.1186/1756-9966-31-97.

84. ParvizHamidi, M., Haddad, G., Ostadrahimi, S., Ostadrahimi, N., Sadeghi, S., Fayaz, S., and Fard-Esfahani, P. (2019). Circulating miR.-26a and miR-21 as biomarkers for glioblastoma multiform. Biotechnol. Appl. Biochem. *66*, 261–265. https://doi.org/10.1002/bab.1707.

85. Hu, X., Chen, D., Cui, Y., Li, Z., and Huang, J. (2013). Targeting microRNA-23a to inhibit glioma cell invasion via HOXD10. Sci. Rep. *3*, 3423. https://doi.org/10.1038/srep03423.

86. Yachi, K., Tsuda, M., Kohsaka, S., Wang, L., Oda, Y., Tanikawa, S., Ohba, Y., and Tanaka, S. (2018). miR-23a promotes invasion of glioblastoma via HOXD10-regulated glial-mesenchymal transition. Signal Transduct. Target. Ther. *3*, 33. https://doi.org/10.1038/s41392-018-0033-6.

87. Huse, J.T., Brennan, C., Hambardzumyan, D., Wee, B., Pena, J., Rouhanifard, S.H., Sohn-Lee, C., le Sage, C., Agami, R., Tuschl, T., and Holland, E.C. (2009). The PTEN-regulating microRNA miR-26a is amplified in high-grade glioma and facilitates gliomagenesis in vivo. Genes Dev. *23*, 1327–1337. https://doi.org/10.1101/gad.1777409.

88. Zhong, F., Huang, T., and Leng, J. (2019). Serum miR-29b as a novel biomarker for glioblastoma diagnosis and prognosis. Int. J. Clin. Exp. Pathol. *12*, 4106–4112.

89. Géczi, D., Nagy, B., Szilágyi, M., Penyige, A., Birkó, Z., Klekner, Á., Jenei, A., and Birkó, Z. (2021). Analysis of Circulating miRNA Profile in Plasma Samples of Glioblastoma Patients. Int. J. Mol. Sci. *22*, 5058. https://doi.org/10.3390/ijms22105058.

90. Rahmati, Y., Alivand, M., and Mollanoori, H. (2021). MiR-330-3p and miR-485-5p as biomarkers for glioblastoma: An integrated bioinformatics and experimental study. Comput. Biol. Chem. *92*, 107458. https://doi.org/10.1016/j.compbiolchem.2021.107458.

91. Yamashita, D., Suehiro, S., Ohtsuka, Y., Ozaki, S., Nishikawa, M., Inoue, A., Kohno, S., Ohue, S., Ohnishi, T., and Kunieda, T. (2021). COT-3 Exosomal microRNA expression signature in blood and cerebrospinal fluid of glioblastoma patients. Neurooncol. Adv. *3*, vi28–vi29. https://doi.org/10.1093/noajnl/vdab159.111.

92. Zhang, H., Wang, J., Wang, Z., Ruan, C., Wang, L., and Guo, H. (2019). Serum miR-100 is a potential biomarker for detection and outcome prediction of glioblastoma patients. Cancer Biomarkers *24*, 43–49. https://doi.org/10.3233/cbm-181416.

93. Akers, J.C., Hua, W., Li, H., Ramakrishnan, V., Yang, Z., Quan, K., Zhu, W., Li, J., Figueroa, J., Hirshman, B.R., et al. (2017). A cerebrospinal fluid microRNA signature as biomarker for glioblastoma. Oncotarget *8*, 68769–68779. https://doi.org/10.18632/oncotarget.18332.

94. Li, H.Y., Li, Y.M., Li, Y., Shi, X.W., and Chen, H. (2016). Circulating microRNA-137 is a potential biomarker for human glioblastoma. Eur. Rev. Med. Pharmacol. Sci. *20*, 3599–3604.

95. Sippl, C., Quiring, A., Teping, F., Schulz-Schaeffer, W., Urbschat, S., Ketter, R., and Oertel, J. (2022). MiRNA-181d Expression Correlates in Tumor versus Plasma of Glioblastoma Patients-the Base of a Preoperative Stratification Tool for Local Carmustine Wafer Use. World Neurosurg. *159*, e324–e333. https://doi.org/10.1016/j.wneu.2021.12.053.

96. Stakaitis, R., Pranckeviciene, A., Steponaitis, G., Tamasauskas, A., Bunevicius, A., and Vaitkiene, P. (2020). Unique Interplay between Molecular miR-181b/d Biomarkers and Health Related Quality of Life Score in the Predictive Glioma Models. Int. J. Mol. Sci. *21*, 7450. https://doi.org/10.3390/ijms21207450.

97. Wu, J., Al-Zahrani, A., Beylerli, O., Sufianov, R., Talybov, R., Meshcheryakova, S., Sufianova, G., Gareev, I., and Sufianov, A. (2022). Circulating miRNAs as

Diagnostic and Prognostic Biomarkers in High-Grade Gliomas. Front. Oncol. *12*, 898537. https://doi.org/10.3389/fonc.2022.898537.

98. Xiao, Y., Zhang, L., Song, Z., Guo, C., Zhu, J., Li, Z., and Zhu, S. (2016). Potential Diagnostic and Prognostic Value of Plasma Circulating MicroRNA-182 in Human Glioma. Med. Sci. Monit. *22*, 855–862. https://doi.org/10.12659/msm.897164.

99. Kouri, F.M., Ritner, C., and Stegh, A.H. (2015). miRNA-182 and the regulation of the glioblastoma phenotype - toward miRNA-based precision therapeutics. Cell Cycle *14*, 3794–3800. https://doi.org/10.1080/15384101.2015.1093711.

100. Lai, N.S., Wu, D.G., Fang, X.G., Lin, Y.C., Chen, S.S., Li, Z.B., and Xu, S.S. (2015). Serum microRNA-210 as a potential noninvasive biomarker for the diagnosis and prognosis of glioma. Br. J. Cancer *112*, 1241–1246. https://doi.org/10.1038/bjc.2015.91.

101. Lan, F., Yue, X., and Xia, T. (2020). Exosomal microRNA-210 is a potentially non-invasive biomarker for the diagnosis and prognosis of glioma. Oncol. Lett. *19*, 1967–1974. https://doi.org/10.3892/ol.2020.11249.

102. Olioso, D., Caccese, M., Santangelo, A., Lippi, G., Zagonel, V., Cabrini, G., Lombardi, G., and Dechecchi, M.C. (2021). Serum Exosomal microRNA-21, 222 and 124-3p as Noninvasive Predictive Biomarkers in Newly Diagnosed High-Grade Gliomas: A Prospective Study. Cancers *13*, 3006. https://doi.org/10.3390/cancers13123006.

103. Manterola, L., Guruceaga, E., Gállego Pérez-Larraya, J., González-Huarriz, M., Jauregui, P., Tejada, S., Diez-Valle, R., Segura, V., Samprón, N., Barrena, C., et al. (2014). A small noncoding RNA signature found in exosomes of GBM patient serum as a diagnostic tool. Neuro Oncol. *16*, 520–527. https://doi.org/10.1093/neuonc/not218.

104. Li, H., Yu, L., Liu, J., Bian, X., Shi, C., Sun, C., Zhou, X., Wen, Y., Hua, D., Zhao, S., et al. (2017). miR-320a functions as a suppressor for gliomas by targeting SND1 and β-catenin, and predicts the prognosis of patients. Oncotarget *8*, 19723–19737. https://doi.org/10.18632/oncotarget.14975.

105. Vojdani, S., Ghaderian, S.M.H., Zali, A., Rakhshan, A., Oraee Yazdani, S., Poursheikhani, A., Bidari Zerehpoush, F., and Sharifi, G. (2021). Altered expression of EGFR and miR-34a derived from serum and tumoral tissue was associated with glioblastoma multiform. Exp. Mol. Pathol. *121*, 104655. https://doi.org/10.1016/j.yexmp.2021.104655.

106. Godlewski, J., Nowicki, M.O., Bronisz, A., Nuovo, G., Palatini, J., De Lay, M., Van Brocklyn, J., Ostrowski, M.C., Chiocca, E.A., and Lawler, S.E. (2010). MicroRNA-451 regulates LKB1/AMPK signaling and allows adaptation to metabolic stress in glioma cells. Mol. Cell *37*, 620–632. https://doi.org/10.1016/j.molcel.2010.02.018.

107. Kim, Y., Roh, S., Lawler, S., and Friedman, A. (2011). miR451 and AMPK mutual antagonism in glioma cell migration and proliferation: a mathematical model. PLoS One *6*, e28293. https://doi.org/10.1371/journal.pone.0028293.

108. Wang, Z.Q., Zhang, M.Y., Deng, M.L., Weng, N.Q., Wang, H.Y., and Wu, S.X. (2017). Low serum level of miR-485-3p predicts poor survival in patients with glioblastoma. PLoS One *12*, e0184969. https://doi.org/10.1371/journal.pone.0184969.

109. Chang, Q., Jiang, Y., Qian, J., Yang, J., Yan, X., and Xue, X. (2018). Advances in exosome-related biomarkers for glioblastoma: Basic research and clinical application. Glioma *1*, 159–167. https://doi.org/10.4103/glioma.glioma_35_18.

110. Peng, J., Liang, Q., Xu, Z., Cai, Y., Peng, B., Li, J., Zhang, W., Kang, F., Hong, Q., Yan, Y., and Zhang, M. (2021). Current Understanding of Exosomal MicroRNAs in Glioma Immune Regulation and Therapeutic Responses. Front. Immunol. *12*, 813747. https://doi.org/10.3389/fimmu.2021.813747.

111. Yang, C., Wang, C., Chen, X., Chen, S., Zhang, Y., Zhi, F., Wang, J., Li, L., Zhou, X., Li, N., et al. (2013). Identification of seven serum microRNAs from a genome.-wide serum microRNA expression profile as potential noninvasive biomarkers for malignant astrocytomas. Int. J. Cancer *132*, 116–127. https://doi.org/10.1002/ijc.27657.

112. Sun, J., Sun, Z., Gareev, I., Yan, T., Chen, X., Ahmad, A., Zhang, D., Zhao, B., Beylerli, O., Yang, G., and Zhao, S. (2021). Exosomal miR-2276-5p in Plasma Is a Potential Diagnostic and Prognostic Biomarker in Glioma. Front. Cell Dev. Biol. *9*, 671202. https://doi.org/10.3389/fcell.2021.671202.

113. Joo Park, H., Hwang, K., Kwon, J.E., Han, J.Y., Park, K.U., Hong, Y.K., and Kim, C.Y. (2022). The effectiveness of circulating micro rnas derived from glioblastoma as a biomarker for early diagnosis and prognosis. Neuro Oncol. *24*, vii8. https://doi.org/10.1093/neuonc/noac209.031.

114. Zhai, F., Chen, X., He, Q., Zhang, H., Hu, Y., Wang, D., Liu, S., and Zhang, Y. (2019). MicroRNA-181 inhibits glioblastoma cell growth by directly targeting CCL8. Oncol. Lett. *18*, 1922–1930. https://doi.org/10.3892/ol.2019.10480.

115. Zhang, Y., Sui, R., Chen, Y., Liang, H., Shi, J., and Piao, H. (2019). Downregulation of miR-485-3p promotes glioblastoma cell proliferation and migration via targeting RNF135. Exp. Ther. Med. *18*, 475–482. https://doi.org/10.3892/etm.2019.7600.

116. Zhao, H., Shen, J., Hodges, T.R., Song, R., Fuller, G.N., and Heimberger, A.B. (2017). Serum microRNA profiling in patients with glioblastoma: a survival analysis. Mol. Cancer *16*, 59. https://doi.org/10.1186/s12943-017-0628-5.

117. Shi, R., Wang, P.Y., Li, X.Y., Chen, J.X., Li, Y., Zhang, X.Z., Zhang, C.G., Jiang, T., Li, W.B., Ding, W., and Cheng, S.J. (2015). Exosomal levels of miRNA-21 from cerebrospinal fluids associated with poor prognosis and tumor recurrence of glioma patients. Oncotarget *6*, 26971–26981. https://doi.org/10.18632/oncotarget.4699.

118. Zhang, Y., Ta, W.W., Sun, P.F., Meng, Y.F., and Zhao, C.Z. (2019). Diagnostic and prognostic significance of serum miR-145-5p expression in glioblastoma. Int. J. Clin. Exp. Pathol. *12*, 2536–2543.

119. Zeng, A., Wei, Z., Yan, W., Yin, J., Huang, X., Zhou, X., Li, R., Shen, F., Wu, W., Wang, X., and You, Y. (2018). Exosomal transfer of miR-151a enhances chemosensitivity to temozolomide in drug-resistant glioblastoma. Cancer Lett. *436*, 10–21. https://doi.org/10.1016/j.canlet.2018.08.004.

120. Bustos, M.A., Rahimzadeh, N., Ryu, S., Gross, R., Tran, L.T., Renteria-Lopez, V.M., Ramos, R.I., Eisenberg, A., Hothi, P., Kesari, S., et al. (2022). Cell-free plasma microRNAs that identify patients with glioblastoma. Lab. Invest. *102*, 711–721. https://doi.org/10.1038/s41374-021-00720-4.

121. Tang, H., Liu, Q., Liu, X., Ye, F., Xie, X., Xie, X., and Wu, M. (2015). Plasma miR-185 as a predictive biomarker for prognosis of malignant glioma. J. Cancer Res. Ther. *11*, 630–634. https://doi.org/10.4103/0973-1482.146121.

122. Zhu, M., Zhao, W., Zhao, H., and Zhang, J. (2019). Diagnostic and prognostic value of microRNA-193b in patients with glioma and its effect on tumor progression. Oncol. Lett. *18*, 4882–4890. https://doi.org/10.3892/ol.2019.10819.

123. Chen, J., Yang, L., and Wang, X. (2017). Reduced circulating microRNA-203 predicts poor prognosis for glioblastoma. Cancer Biomarkers *20*, 521–526. https://doi.org/10.3233/cbm-170335.

124. Yue, X., Lan, F., Hu, M., Pan, Q., Wang, Q., and Wang, J. (2016). Downregulation of serum microRNA-205 as a potential diagnostic and prognostic biomarker for human glioma. J. Neurosurg. *124*, 122–128. https://doi.org/10.3171/2015.1.JNS141577.

125. Hartanto, R.A., Tamba, D.A., Malueka, R.G., and Sutarni, S. (2024). AB071. Predictive value of plasma microRNA-10b and microRNA-21 on chemotherapy toxicity, recurrence, and overall survival in high-grade glioma patients treated with temozolomide. Chin. Clin. Oncol. *13*, AB071. https://doi.org/10.21037/cco-24-ab071.

126. Siegal, T., Charbit, H., Paldor, I., Zelikovitch, B., Canello, T., Benis, A., Wong, M.L., Morokoff, A.P., Kaye, A.H., and Lavon, I. (2016). Dynamics of circulating hypoxia-mediated miRNAs and tumor response in patients with high-grade glioma treated with bevacizumab. J. Neurosurg. *125*, 1008–1015. https://doi.org/10.3171/2015.8.JNS15437.

127. Yin, J., Zeng, A., Zhang, Z., Shi, Z., Yan, W., and You, Y. (2019). Exosomal transfer of miR-1238 contributes to temozolomide-resistance in glioblastoma. EBioMedicine *42*, 238–251. https://doi.org/10.1016/j.ebiom.2019.03.016.

128. Morokoff, A., Jones, J., Nguyen, H., Ma, C., Lasocki, A., Gaillard, F., Bennett, I., Luwor, R., Stylli, S., Paradiso, L., et al. (2020). Serum microRNA is a biomarker for post-operative monitoring in glioma. J. Neuro Oncol. *149*, 391–400. https://doi.org/10.1007/s11060-020-03566-w.

129. Louis, D.N., Perry, A., Wesseling, P., Brat, D.J., Cree, I.A., Figarella-Branger, D., Hawkins, C., Ng, H.K., Pfister, S.M., Reifenberger, G., et al. (2021). The 2021 WHO Classification of Tumors of the Central Nervous System: a summary. Neuro Oncol. *23*, 1231–1251. https://doi.org/10.1093/neuonc/noab106.

130. Hasan, H., Afzal, M., Castresana, J.S., and Shahi, M.H. (2023). A Comprehensive Review of miRNAs and Their Epigenetic Effects in Glioblastoma. Cells *12*, 1578. https://doi.org/10.3390/cells12121578.

131. Roda, D., Veiga, P., Melo, J.B., Carreira, I.M., and Ribeiro, I.P. (2024). Principles in the Management of Glioblastoma. Genes *15*, 501. https://doi.org/10.3390/genes15040501.

132. Choppavarapu, L., and Kandi, S.M. (2021). Circulating microRNAs as Potential Biomarkers in Glioma: A Mini Review. Endocr. Metab. Immune Disord. Drug Targets *21*, 195–202. https://doi.org/10.2174/1871530320666200730230422.

133. Davidson, C.L., Vengoji, R., Jain, M., Batra, S.K., and Shonka, N. (2024). Biological, diagnostic and therapeutic implications of exosomes in glioma. Cancer Lett. *582*, 216592. https://doi.org/10.1016/j.canlet.2023.216592.

134. Leonard, S., Karabegović, I., Ikram, M.A., Ahmad, S., and Ghanbari, M. (2024). Plasma circulating microRNAs associated with blood-based immune markers: a population-based study. Clin. Exp. Immunol. *215*, 251–260. https://doi.org/10.1093/cei/uxad126.

135. Chan, S.F., Cheng, H., Goh, K.K.R., and Zou, R. (2023). Preanalytic Methodological Considerations and Sample Quality Control of Circulating miRNAs. J. Mol. Diagn. *25*, 438–453. https://doi.org/10.1016/j.jmoldx.2023.03.005.

136. Pacia, C.P., Yuan, J., Yue, Y., Xu, L., Nazeri, A., Desai, R., Gach, H.M., Wang, X., Talcott, M.R., Chaudhuri, A.A., et al. (2022). Sonobiopsy for minimally invasive, spatiotemporally-controlled, and sensitive detection of glioblastoma-derived circulating tumor DNA. Theranostics *12*, 362–378. https://doi.org/10.7150/thno.65597.

137. Godoy, P.M., Barczak, A.J., DeHoff, P., Srinivasan, S., Etheridge, A., Galas, D., Das, S., Erle, D.J., and Laurent, L.C. (2019). Comparison of Reproducibility, Accuracy, Sensitivity, and Specificity of miRNA Quantification Platforms. Cell Rep. *29*, 4212–4222.e5. https://doi.org/10.1016/j.celrep.2019.11.078.

138. Vafadar, A., Alavimanesh, S., Babadi, S., Hosseinpour, V., Ehtiati, S., and Savardashtaki, A. (2026). MicroRNA biosensors for cardiovascular disease detection. Clin. Chim. Acta *578*, 120525. https://doi.org/10.1016/j.cca.2025.120525.

139. Fattahi, M., Maghsudlu, M., Razipour, M., Movahedpour, A., Ghadami, M., Alizadeh, M., Khatami, S.H., Taheri-Anganeh, M., Ghasemi, E., Ghasemi, H., et al. (2024). MicroRNA biosensors for detection of glioblastoma. Clin. Chim. Acta *556*, 117829. https://doi.org/10.1016/j.cca.2024.117829.

140. Feng, S., Chen, H., Hu, Z., Wu, T., and Liu, Z. (2023). Ultrasensitive Detection of miRNA via CRISPR/Cas12a Coupled with Strand Displacement Amplification Reaction. ACS Appl. Mater. Interfaces *15*, 28933–28940. https://doi.org/10.1021/acsami.3c03399.

141. Elias, M.G., Hadjiyiannis, H., Vafaee, F., Scott, K.F., de Souza, P., Becker, T.M., and Fatima, S. (2025). The Quest for Non-Invasive Diagnosis: A Review of Liquid Biopsy in Glioblastoma. Cancers (Basel) *17*, 2700. https://doi.org/10.3390/cancers17162700.

142. Wang, K., Kumar, U.S., Sadeghipour, N., Massoud, T.F., and Paulmurugan, R. (2021). A Microfluidics-Based Scalable Approach to Generate Extracellular Vesicles with Enhanced Therapeutic MicroRNA Loading for Intranasal Delivery to Mouse Glioblastomas. ACS Nano *15*, 18327–18346. https://doi.org/10.1021/acsnano.1c07587.

OMTON, Volume *34*

Supplemental information

Circulating miRNAs for glioblastoma monitoring: from biofluid to clinical decision

Arthur Leclerc, Evelyne Emery, and Guénaëlle Levallet

Table S1. Ranked summary of the most consistently validated circulating miRNAs in glioma across the studies included in this review. Supporting studies refer to independent peer-reviewed reports cited in the present review supporting the indicated clinical use of the miRNA in glioma; a higher count reflects greater inter-study reproducibility. ↑ = upregulated in glioma; ↓ = downregulated; Dynamic = variable longitudinal pattern tracking disease activity. CSF, cerebrospinal fluid; MGMT, O^6-methylguanine-DNA methyltransferase. Reference numbers correspond to the main manuscript reference list.

miRNA	Regulation	Biofluid(s)	Main clinical application(s)	Supporting studies	Consistency tier
**miR-21**	↑	Serum, plasma, CSF, exosomes	Diagnostic, prognostic, monitoring	13 (refs 41, 76–82, 112, 113, 123, 124, 126)	High
**miR-222**	↑	Serum, plasma, CSF	Prognostic, monitoring	4 (refs 98, 112, 114, 123)	Moderate–high
**miR-181 family**	↓	Serum, plasma	Prognostic, MGMT correlation, treatment response	3 (refs 83, 103, 117)	Moderate–high
**miR-182**	↑	Plasma, serum	Diagnostic, prognostic	3 (refs 86, 87, 114)	Moderate–high
**miR-124**	↓	Plasma, CSF	Diagnostic	3 (refs 89, 98, 112)	Moderate–high
**miR-128**	↓	Plasma, CSF	Diagnostic	3 (refs 79, 89, 98)	Moderate–high
**miR-210**	↑	Serum, plasma	Diagnostic, prognostic, anti-angiogenic monitoring	2 (refs 84, 85)	Limited
**miR-10b**	↑	CSF, serum	Diagnostic, monitoring	2 (refs 41, 78)	Limited
**miR-34a/b**	↓	Serum, plasma	Diagnostic	2 (refs 101, 102)	Limited
**miR-196b**	↑	CSF	Diagnostic, prognostic	1 (ref 41)	Single-study
**miR-137**	↓	Plasma	Prognostic	1 (ref 99)	Single-study
**miR-100**	↓	Plasma	Prognostic	1 (ref 100)	Single-study
**miR-223**	Dynamic	Serum	Longitudinal monitoring; recurrence vs. pseudo-progression	1 (ref 126)	Single-study
**miR-320e**	Dynamic	Serum	Longitudinal monitoring; recurrence vs. pseudo-progression	1 (ref 126)	Single-study
**miR-1238 (exosomal)**	↑	Exosomes	Temozolomide resistance	1 (ref 125)	Single-study
